# Supplementary material for: Wearable intelligent throat enables natural speech in stroke patients with dysarthria
Source: Nat Commun. 2026 Jan 19;17:293. doi: 10.1038/s41467-025-68228-9 (PMC12816716; doi:10.1038/s41467-025-68228-9)
Supplement: Supplementary file 1 — Supplementary Information [file 41467_2025_68228_MOESM1_ESM.pdf]

Supplementary Information

Wearable intelligent throat enables natural speech in stroke patients with dysarthria

The supplementary information file includes:

Supplementary Text
Supplementary Figures 1 to 21
Supplementary Tables 1 to 9
Supplementary References

Supplementary Text

Supplementary Note 1: Background and motivation of this study

What is dysarthira and why it is a significant issue?

Dysarthria, a motor speech disorder, significantly impacts patients' quality of life, hindering their ability to communicate and leading to complex physical and psychological challenges. Originating from neuromuscular impairments, dysarthria affects the voluntary control of muscles involved in speech, including the laryngeal, pharyngeal, and facial muscles. This disorder spans across various neurological conditions such as stroke, ALS, Parkinson’s disease, multiple sclerosis, and traumatic brain injuries. Notably, dysarthria occurs in approximately 20–30% of stroke patients, 80–90% of ALS patients, and 60–70% of those with Parkinson's disease, making it a prevalent issue across these populations. These impairments disrupt the nervous system's coordination of speech-related muscle movements, resulting in challenges with articulation, phonation, and resonance, which severely limits effective communication [1, 2, 3, 4].

Beyond communication barriers, dysarthria poses broader consequences that affect both the physical and mental well-being of patients. Social isolation is a prevalent issue, as individuals with dysarthria often struggle to express their thoughts and emotions, leading to a sense of disconnection from those around them. This isolation can create a compounding effect, contributing to heightened levels of anxiety and depression [5, 6]. Furthermore, communication challenges hinder rehabilitation by making it difficult for patients to convey their needs and responses during therapy sessions, which is crucial for personalized adjustments in treatment. This lack of effective feedback can compromise therapeutic outcomes, potentially placing additional physiological strain on patients as they navigate rehabilitation with limited adaptive support [7, 8].

To alleviate these multifaceted challenges, there is an urgent need for augmentative and alternative communication (AAC) technology that enables fluid and contextually rich communication in diverse settings, helping patients restore a sense of connection and control. Effective communication solutions can improve quality of life, reduce mental health risks, and enhance the efficacy of rehabilitation, ultimately promoting holistic well-being for individuals affected by dysarthria.

BCIs achieved remarkable success in complete paralysis cases, yet not enough for broader dysarthria applications

Brain-computer interface (BCI)-based AAC systems are among the most advanced approaches developed for individuals with severe communication impairments. These systems are particularly effective for patients experiencing near-total paralysis, such as those with locked-in syndrome or advanced stages of ALS, where voluntary control over the facial, laryngeal, and other speech-related muscles is completely lost. By bypassing the motor system entirely, BCIs translate brain activity into direct communication output, often through technologies like EEG, ECoG, or even implanted electrodes. For patients with no means of physical communication, BCI-based systems have opened critical avenues for re-engaging with the world, restoring a level of independence and connection that would otherwise be inaccessible [9, 10].

However, despite their transformative potential, BCI-based AAC systems come with significant limitations, especially in terms of accessibility and user-friendliness. First, these systems are often complex and cumbersome, requiring substantial technical setup, ongoing calibration, and expert supervision, which limits their usability in day-to-day environments outside of clinical or highly controlled settings. Additionally, the need for electrodes and the relatively invasive nature of some setups contribute to a lack of portability and comfort, rendering BCI systems impractical for routine, non-hospital use. This limited practicality is compounded by the often substantial cost and the invasive procedures associated with implant-based BCI solutions, making them inaccessible to many who might benefit from an alternative, less intrusive approach.

For patients who retain some voluntary control over facial or laryngeal muscles—an ability present in most dysarthria cases—the limitations of BCI systems highlight a need for more intuitive, accessible solutions that can integrate seamlessly into daily life without sacrificing portability or comfort. For individuals with complete neuromuscular loss, such as advanced ALS or locked-in syndrome patients, brain-computer interface (BCI)-based AAC systems remain the primary alternative, as they directly decode neural activity without requiring any muscle movement. However, BCI systems are often invasive, require complex calibration, and have limited portability, making them less accessible for daily communication. In contrast, the IT system is specifically designed for patients with moderate dysarthria, where residual laryngeal or facial muscle activity enables real-time, non-invasive silent speech decoding.

Wearable silent speech systems: progress and persistent gaps

Wearable silent speech systems represent a promising, non-invasive alternative to BCIs for patients with moderate dysarthria who retain partial neuromuscular control over speech-related muscles. These systems capture subtle, non-acoustic signals, such as muscle vibrations or electrophysiological signals generated through silent articulation, offering a portable and user-friendly solution for those unable to communicate verbally. Unlike BCIs, wearable silent speech systems allow for relatively seamless integration into daily life, bringing potential for greater accessibility and comfort [11-15].

However, despite these advances, wearable silent speech systems still face three significant gaps that limit their ability to deliver truly natural and expressive communication:

1. Lack of zero-delay expression: Current wearable silent speech systems often rely on fixed time windows (e.g., 1–3 seconds) for word decoding. For example, Kim *et al.* utilized a 2s time window in decoding a 100 words dataset [13], and Tang *el al.* utilized a 3s time window to decode a 30 words dataset [14]. This timing requires users to complete each word within a set interval and pause before continuing, leading to fragmented and artificial expression. In real-world communication, fluency is crucial, as it allows for spontaneous, uninterrupted conversation that reflects natural speaking patterns. Without zero-delay expression, users must adjust their speech to the constraints of the device, resulting in an unnatural and often frustrating communication experience.

2. Limited emotional and logical coherence: Another major limitation is the lack of emotional expressiveness and contextual coherence in the sentences generated by current systems. Natural speech is more than a series of words; it conveys emotions, intonations, and logical transitions

that help the listener understand the speaker's intent. Without the ability to capture these nuances, wearable silent speech systems fail to provide a complete and effective communication solution. However, all current silent speech systems can only decode fixed words or sentences, lacking in-depth analysis of emotions and logic [11-15].

3. Restricted accessibility for patient populations: Finally, the accessibility of wearable silent speech systems for a broad range of patients remains limited. Most current systems are tested primarily on healthy individuals, with limited clinical validation among dysarthric patients. Yang *et al.* were the first to test their developed silent speech system on a patient with speech impairment (one post-laryngectomy patient). However, while the system achieved over 99% accuracy in healthy individuals, this accuracy dropped sharply to 90% when transferred to the patient [11]. Without extensive testing and refinement tailored to patients with varying neuromuscular abilities, these silent speech devices may not meet the unique needs of dysarthria patients, especially those with complex neurological conditions. This gap in accessibility underscores the need for more inclusive designs and expanded clinical trials to better serve the dysarthria population.

The intelligent throat: bridging key gaps in wearable silent speech systems

Therefore, we developed the Intelligent Throat (IT) system to address these critical issues, enabling zero-delay expression, enhancing emotional and logical coherence in generated sentences, and expanding accessibility for a broader range of dysarthric patients. By integrating flexible sensing, electronics, and artificial intelligence technologies, the IT system provides an intuitive, real-time communication platform specifically designed for stroke patients with dysarthria, thereby enhancing their social engagement, independence, and quality of life, and helping them reconnect with the world around them on their own terms. Below is a Supplementary Figure howing the comparison between the IT system and state-of-the-art wearable silent speech systems.

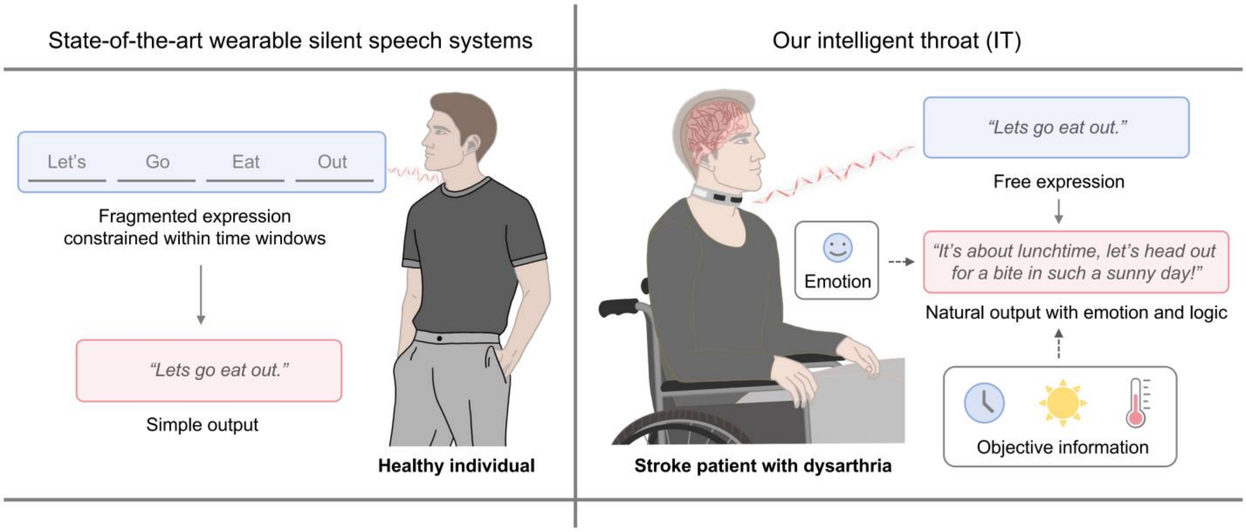

Supplementary Note Figure: Comparison between the IT system and state-of-the-art wearable silent speech systems.

Supplementary Note 2: The latency analysis of the IT system

This note provides an in-depth latency analysis for each component within the IT system. Key elements include PCB transmission, ML computation, LLM generation, and text-to-speech (TTS) synthesis. While the IT system allows for “zero-delay expression,” providing uninterrupted and continuous speech without requiring pauses for word segmentation, it does not fully eliminate communication delay due to waiting for sentence completion. This inherent waiting delay, marked by detecting blank tokens at the end of a sentence, is common in silent speech systems and unavoidable for coherent sentence synthesis. Below, we quantify each source of latency based on theoretical and empirical estimations.

1. PCB transmission latency

The IT system’s PCB facilitates the acquisition and transmission of silent speech and carotid pulse signals. The transmission latency includes data acquisition and data transmission components:

Data acquisition delay: This delay is based on the analog-to-digital converter (ADC) clock and sampling period, yielding a data acquisition delay of **173.33 μs**.

Data dransmission delay: Calculated based on the baud rate of the serial communication port and the data volume, the data transmission delay is approximately **26.04 μs**.

Thus, the total PCB transmission latency is minimal, at **199.37 μs** (or approximately **0.2 ms**).

2. Machine learning model computation latency

The machine learning latency in the IT system arises primarily from the token decoding model, which processes silent speech signals, and the emotion decoding model, which processes carotid pulse signals for emotional context. The token decoding model has a larger computational demand, with a requirement of **0.738 GFLOPs** (student model, 1D ResNet-18), while the emotion decoding model’s computation load is considerably smaller. Given the multi-threading capabilities of modern CPUs, the emotion decoding model can execute concurrently with the token decoding model, effectively making its latency negligible in the overall system performance. Therefore, we focus on estimating the token decoding model’s latency.

Token decoding model

Assuming a typical CPU with a processing efficiency of **10 GFLOPs/ms** (common in modern, low-power multi-threaded processors), the estimated computation time for the token decoding model is:

Token Decoding Model Latency: With **0.738 GFLOPs** required, the processing time on a 10 GFLOP/ms-capable CPU is approximately **0.0738 ms**.

This minimal processing time enables real-time, efficient token decoding, making it feasible for the IT system’s continuous, delay-free expression.

3. LLM generation latency

For a lightweight GPT-4-based API, the sentence generation time typically ranges from **100-150 ms**, depending on context length. For this analysis, we use an average LLM generation delay of **125 ms**.

This latency, while relatively longer than PCB and ML computation delays, remains acceptable for real-time interaction, as it occurs only at the end of a sentence and does not impact the continuous speech decoding process.

4. Text-to-speech (TTS) synthesis and playback latency

Once the LLM generates a sentence, the system uses a TTS model to produce a synthesized audio output matching the user's natural voice. The TTS synthesis and playback latency involve:

TTS model inference delay: With a lightweight, open-source TTS model (such as Tacotron 2 or FastSpeech, here we utilized ByteDance Volcano Engine TTS) capable of real-time synthesis, generating a sentence (around 10 words) takes approximately **150-200 ms**.

Audio playback delay: Standard audio playback latency is negligible, typically **<10 ms** on most devices.

Thus, the TTS synthesis and playback latency totals approximately **160-210 ms**, with **180 ms** as the average estimate.

Total latency summary

PCB transmission (including BLE communication): ~0.2 ms

ML computation (token decoding model): ~0.0738 ms

LLM generation: ~125 ms

TTS synthesis and playback: ~200 ms

The combined theoretical latency of the IT system is approximately **325.27 ms**. In practical tests, however, an average delay of **~1s** was observed between the end of the user's silent expression and the playback of the synthesized sentence. This observed latency accounts for a redundancy measure, where the system waits for the detection of five consecutive blank tokens to confirm sentence completion, enhancing real-world stability.

While the ML model's computation latency is nearly negligible compared to LLM generation and TTS synthesis, optimizing computational power remains essential, especially as future deployments target edge computing devices. Knowledge distillation is therefore vital, compressing the ML model to reduce both computational load and energy consumption, making the system feasible for practical, long-term wearable applications.

Zero-delay expression vs. zero-delay communication

The IT system introduces "zero-delay expression," allowing users to communicate naturally and continuously without pauses or fixed time windows for word segmentation. This high-resolution token segmentation enables a seamless flow of speech, setting a new benchmark for wearable silent speech systems. However, "zero-delay communication" is not fully attainable with SSI systems, as decoding only begins once the user has finished speaking. This inherent delay stems from the need for the silent speech action itself to complete before generating the strain signals required for processing. Unlike systems that might anticipate input, SSI-based approaches naturally rely on the completion of silent articulation, creating a trade-off between real-time, intuitive user interaction and this fundamental latency. Despite this structural limitation, our IT

system optimally balances continuous expression with the brief delay required for sentence decoding, establishing a powerful and practical tool for dysarthria patients. By enabling expressive, natural interaction, the IT system reintroduces ease and fluidity into daily communication.

Supplementary Note 3. Hardware-level advances of the Intelligent Throat (IT) system compared with the previous single-channel smart choker in our lab.

The previous generation of the smart choker [14] consisted of a single strain-sensing channel positioned at the laryngeal region to capture articulatory muscle movements associated with silent speech. In contrast, the current IT introduces both hardware and system-level innovations to enable intelligent expansion and emotion-aware communication.

1. Multichannel architecture.

To incorporate emotion decoding, we added an additional sensing channel aligned with the carotid artery to capture pulse signals related to autonomic emotional changes. The new dual-channel layout thus simultaneously records silent speech strain and pulse dynamics, supporting the dual-task operation of word decoding and emotion inference.

2. Crosstalk mitigation via strain-isolation design.

Because the carotid and laryngeal regions experience coupled vibrations, mechanical crosstalk was a critical issue. Beyond digital filtering, we introduced a polyurethane acrylate (PUA) strain isolation ring printed around each channel to prevent transverse strain propagation. This concept and printing strategy were adapted from our lab's previous work on localized strain isolation [18]. The resulting multilayer sensor stack substantially improves signal separation between the two sensing sites (see Fig. S2).

3. Compact wireless integration.

The previous system [14] relied on a benchtop potentiostat connected to the sensor via copper adhesive tapes, which limited wearability and mobility. The IT system replaces this configuration with a bespoke wireless PCB that integrates low-noise analog front-end circuits, on-board ADC, BLE transmission module, and battery management. The textile sensors are interfaced to the PCB through lightweight snap connectors (see Figs. S4, S5, and S15), forming a fully wearable, self-contained platform suitable for real-time operation.

Collectively, these advances reduce wiring complexity, enhance signal quality, and enable simultaneous silent-speech and emotion decoding in a compact, user-friendly form factor.

Supplementary Note 4. Prompt design and examples for the Token Synthesis Agent (TSA) and Sentence Expansion Agent (SEA)

This note provides the full prompt templates and reasoning examples used for the two LLM-based components in this study, which together reconstruct and expand the user's silently mouthed expressions.

Background and setup

In this study, the vocabulary used for silent speech decoding comprises 47 distinct words, each assigned a numerical label from 1 to 47, while label 0 corresponds to blank tokens representing silent or transitional frames.

This label – word mapping was used as a retrieval-augmented grounding (RAG) source and provided to both the TSA and SEA to ensure consistent reference during token synthesis and sentence generation (Supplementary Table 2). The LLM agents were deployed using the GPT-4o-mini API.

The following example illustrates the full workflow of token-to-sentence generation using the input phrase "We go hospital", which the system expands into a personalized and contextually rich sentence:

"Even though it's getting a bit late, I am still feeling comfortable — can we go to the hospital now?"

This example is divided into two stages:

1. Token Synthesis Agent (TSA) — performs token aggregation and word reconstruction.

2. Sentence Expansion Agent (SEA) — expands the basic sentence using emotion and contextual information.

Token Synthesis Agent (TSA)

Token decoding input:

The token decoding network outputs a sequence of token predictions corresponding to articulatory gestures:

[0, 0, 1, 3, 3, 10, 10, 10, 10, 10, 0, 9, 9, 9, 9, 9, 18, 9, 9, 9, 9, 9, 9, 0, 0, 0, 0, 0]

Here, 0 represents blank or silent frames, and 1 is a misclassified label that should belong to the word "We" (label 3).

The ground-truth word labels for this utterance are:

[3, 10, 9] → "We go hospital"

The prompt used is shown below:

You are a Token Synthesis Agent (TSA) responsible for reconstructing coherent
word sequences from noisy token label streams generated by a wearable silent-
speech decoding model.

Each number in the sequence corresponds to a token label:

• Labels 1–47 → valid vocabulary words
• Label 0 → blank / silence / transitional token

Your goal is to output a clean, ordered sequence of word labels that faithfully represents the user's intended speech. Follow the rules below exactly.

---

1. Merge consecutive identical tokens

If the same non-zero label appears repeatedly (e.g., 3 3 3 3 3),
treat the entire run as one occurrence of that label (3).
This merges sustained articulations that the decoder has oversplit.

---

2. Remove blank tokens (label 0)

Delete all zeros. They represent silence or boundary transitions.

---

3. Remove brief noisy fragments

If a non-zero token occurs only once between two stable regions,
for example "… 3 3 3 1 3 3 3 …", replace that single token with
the surrounding majority label (1 → 3).
If a single token occurs between different labels and has no clear
majority context, remove it as noise.
After correction, re-merge identical tokens if necessary.

---

4. Contextual correction by majority voting

If a non-zero token differs from both its left and right neighbors,
and both neighbors are identical (A B A pattern),
correct the middle token B → A.
When uncertainty remains, inspect a 3–5-token temporal window and keep
the label that dominates the neighborhood.
If no rule clearly applies, keep the original label.

---

5. Apply empirical token-count constraints

Use these approximate token-count expectations when merging or splitting:
• 1–2 tokens → short words (e.g., “we”, “go”)
• 3–6 tokens → medium words (e.g., “hospital”, “morning”)
• ≥7 tokens → long or compound expressions
If a cluster exceeds the expected range for its word type,
check for over-merging and split accordingly.

---

6. Output requirements

• Produce a cleaned list of word labels in order (e.g., [3, 10, 9]).
• Optionally map them to words using the provided label–word dictionary.
• Do not paraphrase, add, or reorder content.
• Output must remain strictly faithful to the decoded sequence after
noise removal and contextual correction.

Few-shot reasoning examples

Example 1
Input: [0, 0, 0, 3, 1, 3, 45, 45, 45, 45, 45,
0, 47, 47, 47, 47, 47, 47, 47, 47, 0, 0, 0]
→ Remove blanks, correct 1→3, merge duplicates
Output: [3, 45, 47] → “We phone contact”

Example 2
Input: [0, 0, 5, 5, 6, 5, 5, 0]
→ Single 6 between 5s → replace 6→5 → [5] → “want”

Example 3
Input: [0, 5, 5, 5, 5, 7, 7, 7, 0, 0, 0]
→ Two stable clusters → [5, 7] → “want eat”

Example 4
Input: [0, 0, 2, 2, 2, 2, 2, 0, 15, 15, 15, 15, 15, 27, 15, 17, 17, 17, 17, 0, 10, 16, 16, 16, 16, 0]
→ Remove blanks, correct 27→17, correct 10→16, merge duplicates
Output: [2, 15, 17, 16] → “My eyes not comfortable”

Final rule

If no correction rule clearly applies, retain the original label rather than inventing a new one. Your task is denoising and consolidation, not rewriting.

Sentence Expansion Agent (SEA)

You are a Sentence Expansion Agent (SEA) designed to help dysarthric stroke patients express natural, emotionally coherent sentences from concise base phrases. Your role is to expand the patient's short silent-speech output into a complete sentence that sounds natural, empathetic, and contextually appropriate.

---

1. Background

You will be given:
• A base sentence decoded from the patient's silent-speech signal.
• The patient's current emotion label.
• Objective contextual information, including time, temperature, and weather.

Your goal:
Generate one complete, fluent, and emotionally aligned sentence that reflects the patient's intention. Maintain the core meaning of the base sentence while gently enriching it with natural emotional tone and contextual information.

---

2. Patient's typical speaking style examples

The following examples illustrate the patient's usual tone and phrasing style. These represent how the patient naturally expresses feelings and requests:

Example Style 1:
"It's getting kind of late and the air feels a bit cool, but I still feel okay — maybe we can take a short walk before heading back?"

Example Style 2:
"I'm feeling a bit tired and frustrated, but I'm trying to stay calm and get through it."

Example Style 3:

“The weather isn’t great tonight, but I still want to finish what I was doing before I rest.”

Your generated output should follow this tone, reflecting the patient’s habitual way of expressing needs and emotions.

---

3. Input for current generation

Base sentence: “We go hospital.”
Emotion label: “Frustrated”
Objective context: “10:20 pm, 18 °C, Cloudy”

---

4. Output requirements

• Expand the base sentence into one fluent, contextually enriched sentence.
• Incorporate the emotion (frustrated) and context (time and weather) subtly.
• Keep the overall tone as if spoken by the patient.
• Preserve the core meaning (intention to go to the hospital).
• Avoid over-generation or changing the factual meaning.

These prompt templates and examples are provided in full to ensure reproducibility and transparency of the LLM-based post-processing pipeline.

Supplementary Figure 1: Schematic of the printing process. The number of prints refers to the number of cycles the substrate is printed for. During each cycle, the silk frame is lowered to the substrate, the flood blade travels backward to spread the ink, the squeegee travels to print, and the silk frame is raised to leave the substrate.

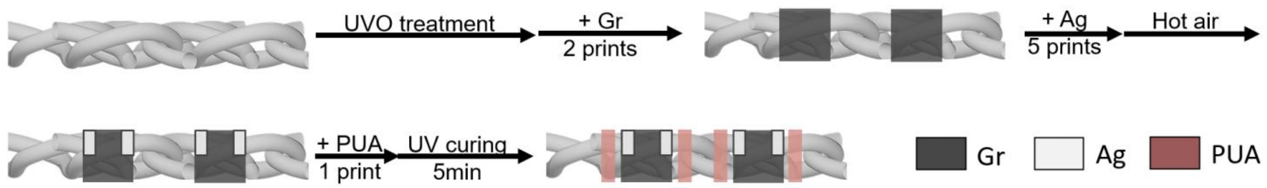

Supplementary Figure 2: Finite element analysis (FEA) simulation for strain distribution of the smart choker under 40% of uniaxial tensile.

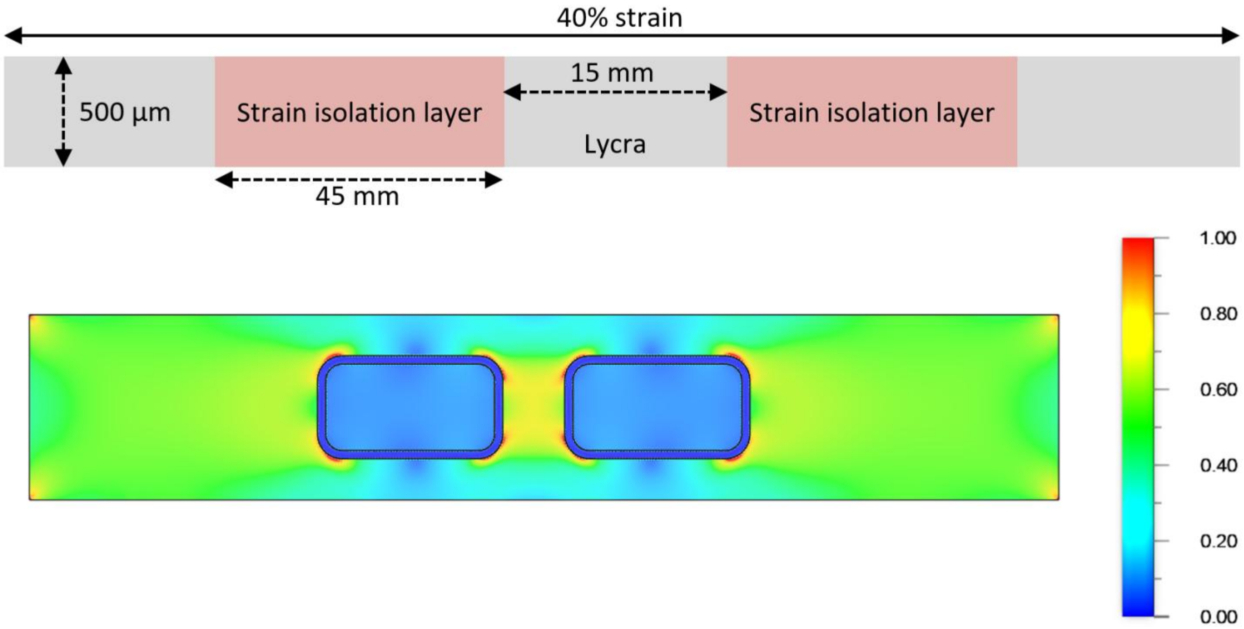

Supplementary Figure 3: Sweat environment test on the sensing area of the artificial throat. According to ISO 3160-2 standard, we prepared artificial sweat solution consisting of 20 g/L sodium chloride (NaCl), 17.5 g/L ammonium chloride (NH4Cl), 5 g/L urea, 2.5 g/L acetic acid (CH3COOH), 15 g/L lactic acid, 80 g/L sodium hydroxide (NaOH) for pH adjustment, and deionized water. The pH of the solution was adjusted to 4.7 using NaOH. Sweat volumes were calculated based on the surface area of the smart garment's sensing area, with minimum (0.32 mg/cm²/min) and maximum (2.7 mg/cm²/min) sweat rates applied over a 10-minute period [16]. The calculated artificial sweat was evenly distributed across the sensing area using a pipette. The artificial throat was in the environment of 16 °C, 63% RH for the duration of the test.

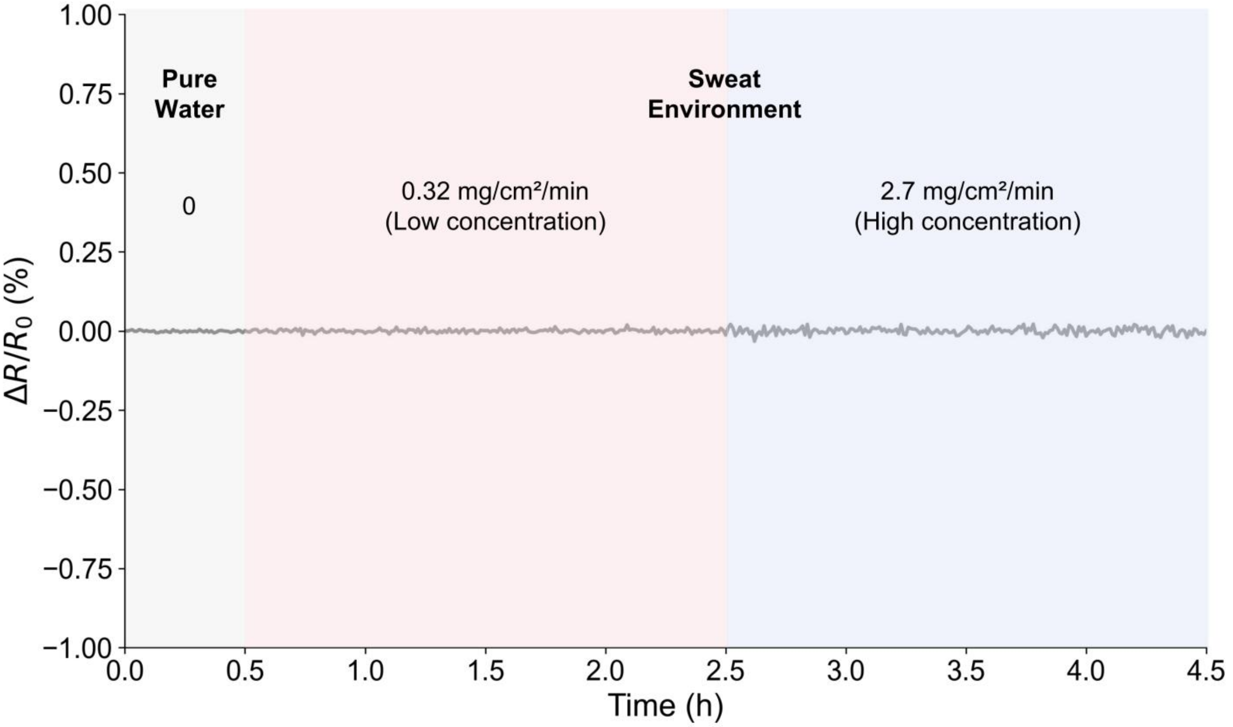

Supplementary Figure 4: Circuit schematic of IT's electronic system.

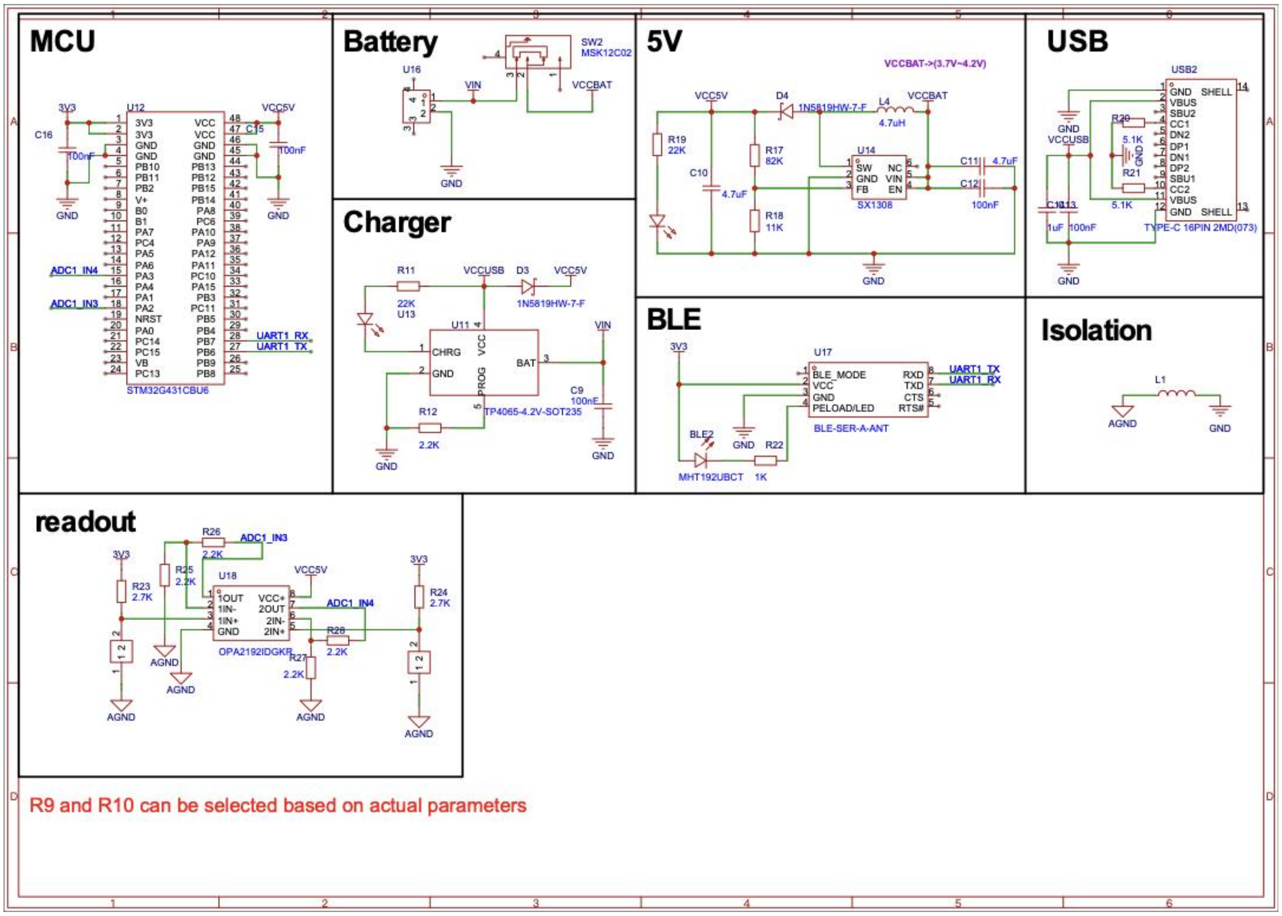

Supplementary Figure 5: The miniaturized PCB of IT system.

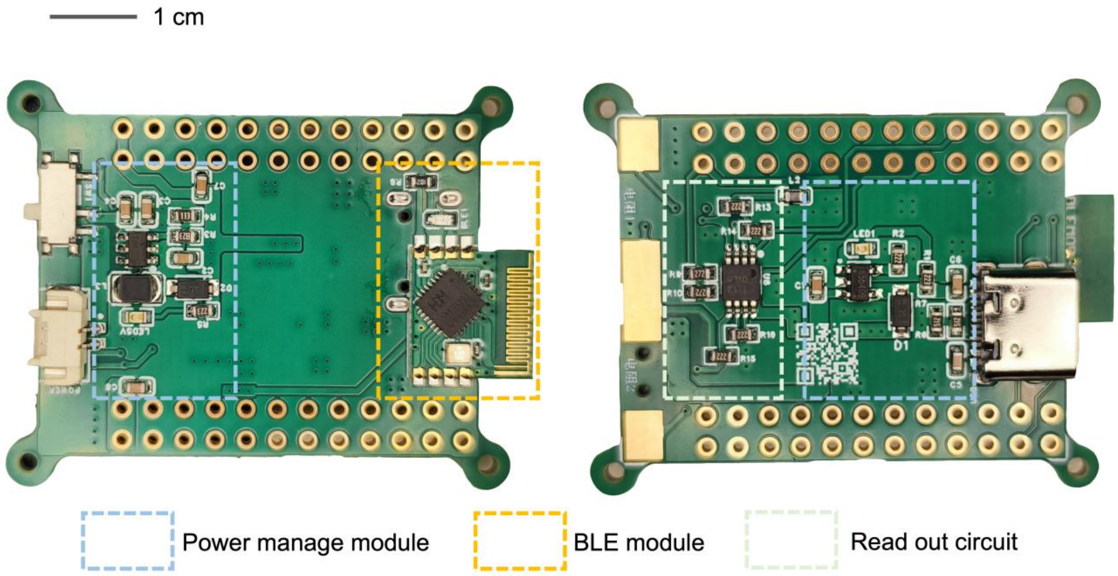

Supplementary Figure 6: Time-frequency spectrogram display of silent speech signals.

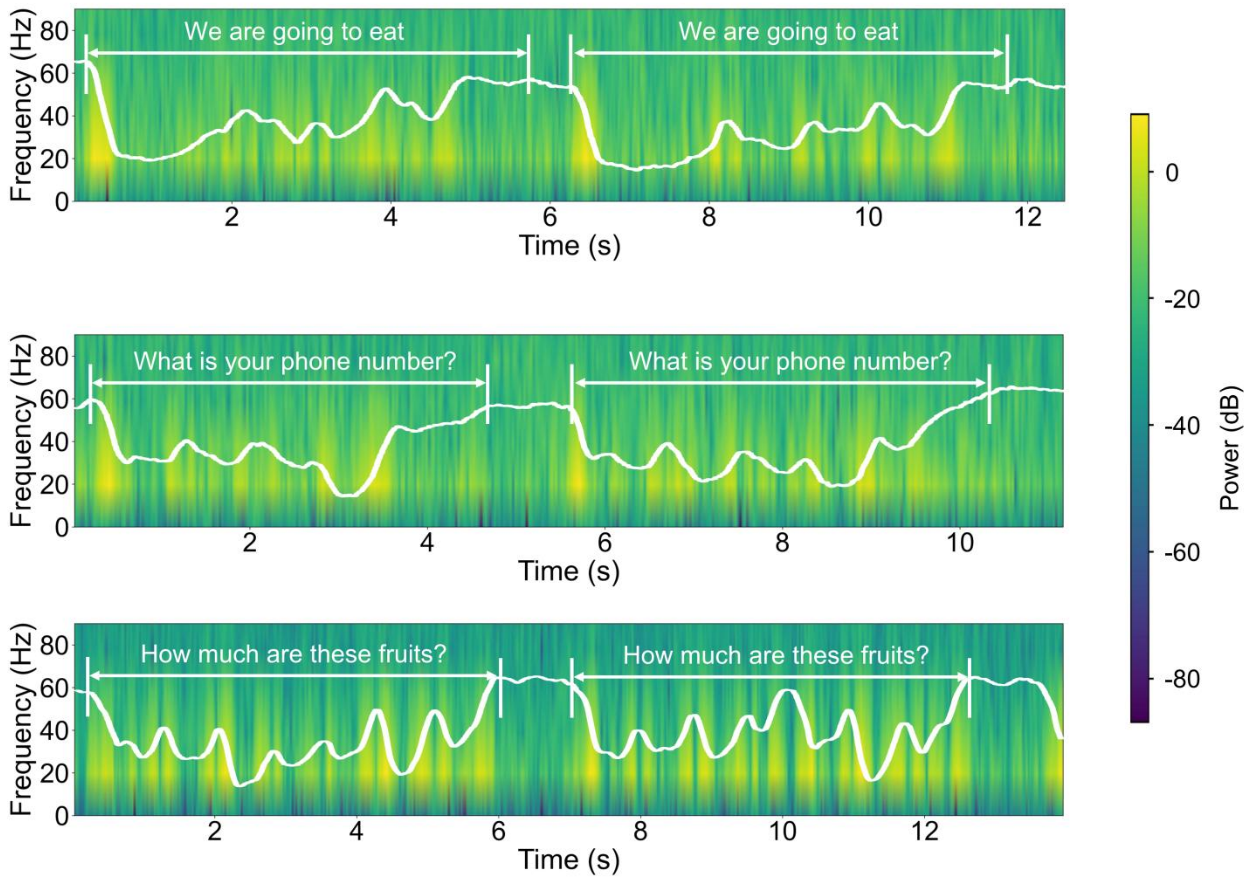

Supplementary Figure 7: Time-frequency spectrogram display of pulse signals.

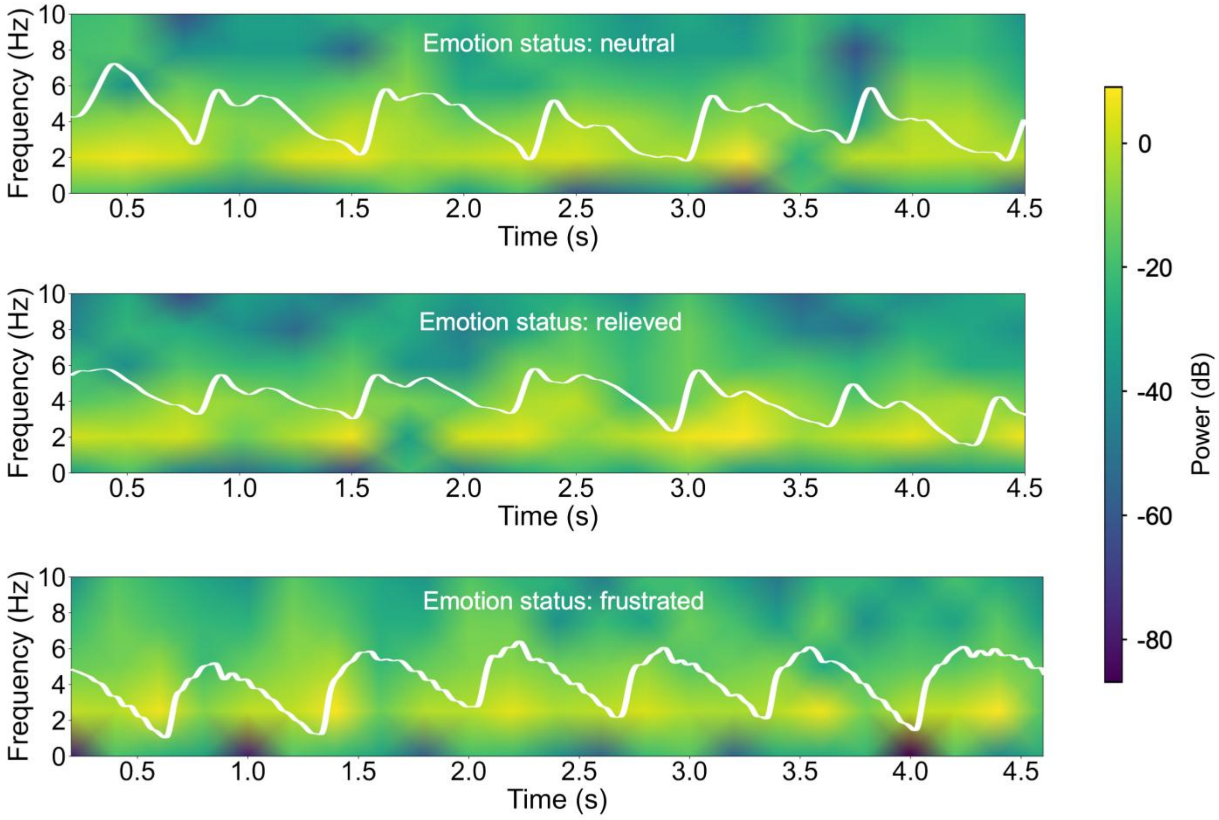

Supplementary Figure 8: The optimal parameters and training curves of the final token decoding model (with 1D ResNet-101 as the teacher model and 1D ResNet-18 as the student model). After pre-training on healthy individuals’ data, fine-tuning on patients’ data, and transferring to the more computationally efficient 1D ResNet-18 via knowledge distillation, the model achieved a token decoding accuracy of 91.3%. Notably, in the pre-training stage, we trained for only 10 epochs; although continued training could further reduce the loss, testing revealed that a lower pre-train loss did not improve the final performance. We believe this is because overtraining during the pre-training phase may lead the model to overfit to healthy individuals' silent speech features, thereby reducing its generalizability.

a

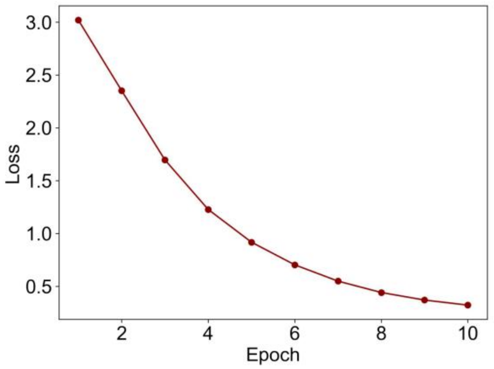

Process	Pre-train
Criterion	Cross-entropy
Early_stop threshold	1e-4
Optimizer	Adam
Patience	10
Batch_size	128
Learning rate	1e-4
Epoch	10

b

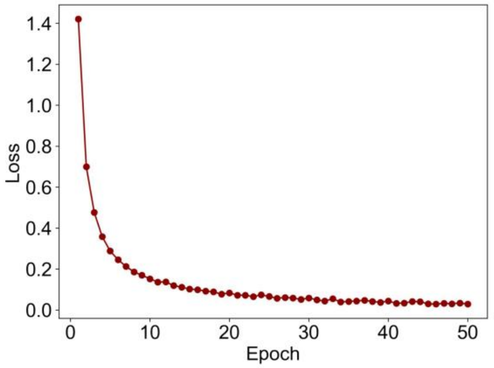

Process	Fine-tune
Criterion	Cross-entropy
Early_stop threshold	1e-4
Optimizer	Adam
Patience	10
Batch_size	128
Learning rate	1e-4
Epoch	50

c

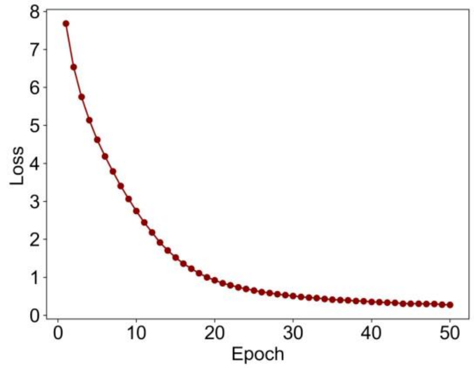

Process	Distillation
Criterion	hybrid distillation loss
Early_stop threshold	1e-4
Optimizer	Adam
Patience	10
Batch_size	128
Learning rate	7e-5
Dropout	0.2

Supplementary Figure 9: The optimal parameters and training curves of other models in the comparative experiments.

1D ResNet-18 (Accuracy: 83.5%, Flops: 0.738G)

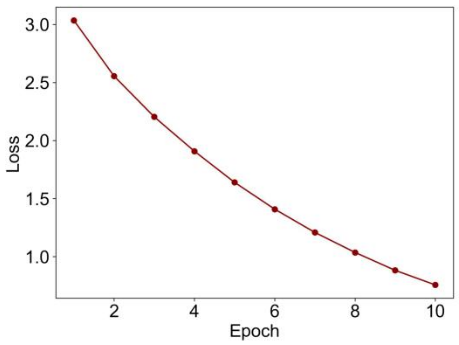

Process	Pre-train
Criterion	Cross-entropy
Early_stop threshold	1e-4
Optimizer	Adam
Patience	10
Batch_size	128
Learning rate	2e-4
Epoch	10

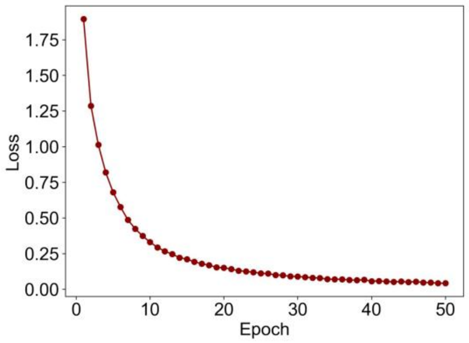

Process	Fine-tune
Criterion	Cross-entropy
Early_stop threshold	1e-4
Optimizer	Adam
Patience	10
Batch_size	128
Learning rate	2e-4
Epoch	50

1D ResNet-34 (Accuracy: 87.8%, Flops: 1.51G)

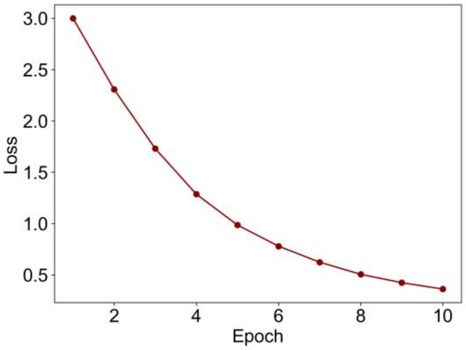

**Process**	**Pre-train**
Criterion	Cross-entropy
Early_stop threshold	1e-4
Optimizer	Adam
Patience	10
Batch_size	128
Learning rate	1e-4
Epoch	10

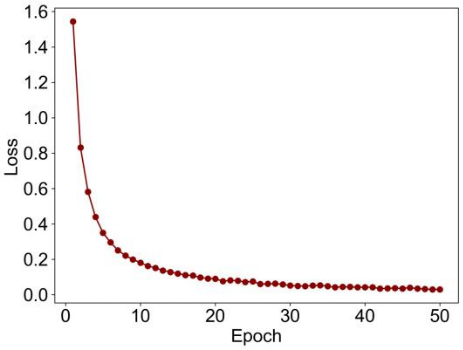

**Process**	**Fine-tune**
Criterion	Cross-entropy
Early_stop threshold	1e-4
Optimizer	Adam
Patience	10
Batch_size	128
Learning rate	9e-5
Epoch	50

1D ResNet-50 (Accuracy: 90.2%, Flops: 1.510G)

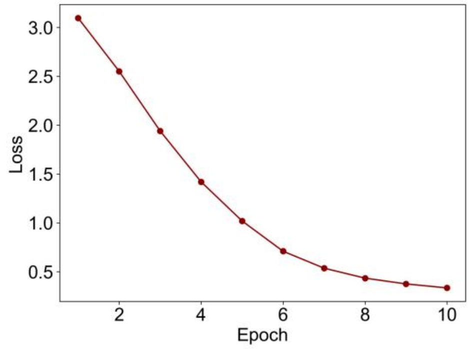

Process	Pre-train
Criterion	Cross-entropy
Early_stop threshold	1e-4
Optimizer	Adam
Patience	10
Batch_size	128
Learning rate	1e-4
Epoch	10

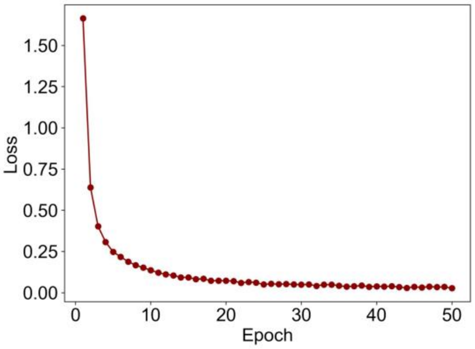

Process	Fine-tune
Criterion	Cross-entropy
Early_stop threshold	1e-4
Optimizer	Adam
Patience	10
Batch_size	128
Learning rate	1e-4
Epoch	50

Transformer (Accuracy: 90.2%, Flops: 5.673G, Without context augmentation)

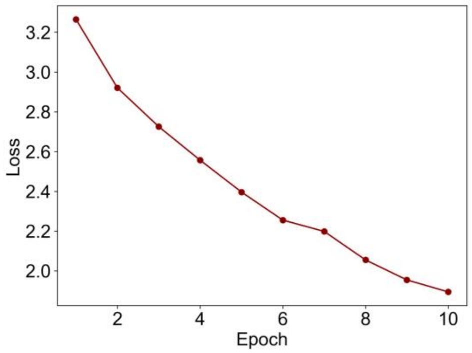

Process	Pre-train
Criterion	Cross-entropy
Early_stop threshold	1e-4
Optimizer	Adam
Patience	10
Batch_size	128
Learning rate	2e-4
Epoch	10

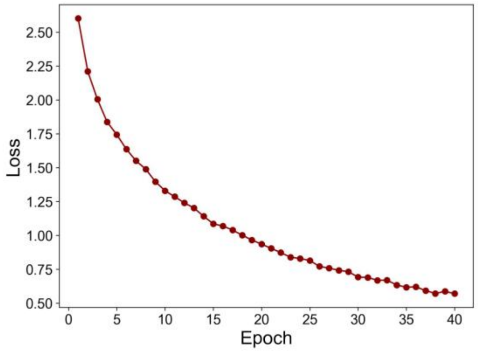

Process	Fine-tune
Criterion	Cross-entropy
Early_stop threshold	1e-4
Optimizer	Adam
Patience	10
Batch_size	128
Learning rate	3e-4
Epoch	40

1D VGG-16 (Accuracy: 85.8%, Flops: 1.253G)

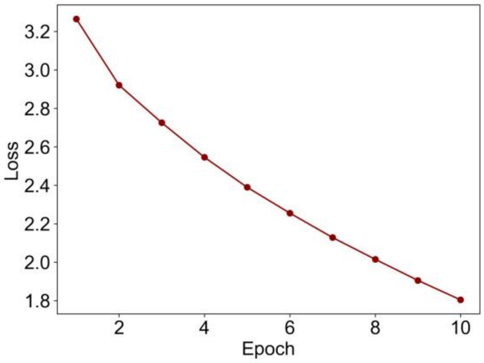

Process	Pre-train
Criterion	Cross-entropy
Early_stop threshold	1e-4
Optimizer	Adam
Patience	10
Batch_size	128
Learning rate	1e-4
Epoch	10

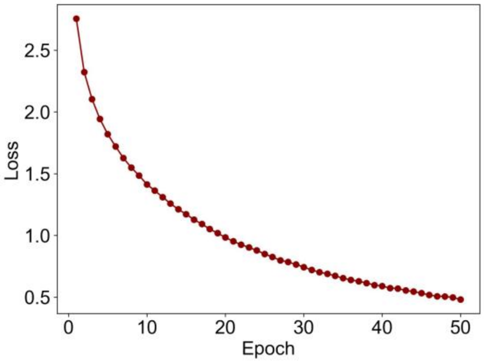

Process	Fine-tune
Criterion	Cross-entropy
Early_stop threshold	1e-4
Optimizer	Adam
Patience	10
Batch_size	128
Learning rate	7e-5
Epoch	50

Supplementary Figure 10: Attention map (SmoothGrad) visualizes the contribution distribution of signals within token-combined samples for classification. It can be observed that the token decoding model pays more attention on the signal area at the end of the sample (current token) when making classification decisions, while also giving a relatively evenly distributed but lower level of attention to signals from other parts (context tokens). This indicates that when determining the class of the current token, the model primarily relies on the signal information of the current token, while also taking into account the preceding information from the context tokens [17].

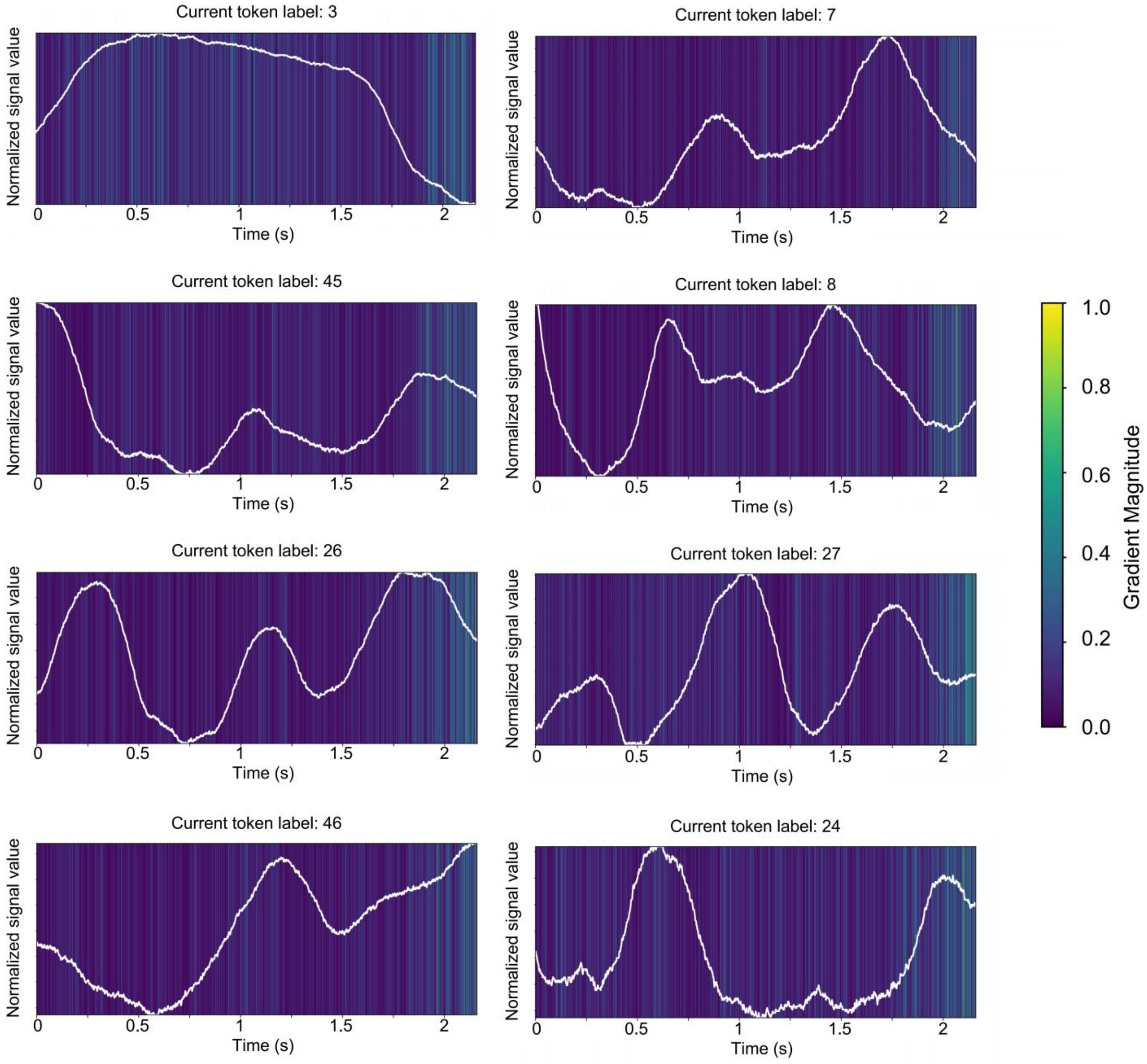

Supplementary Figure 11: SHAP plot illustrating the contributions of different pulse signal frequency bands to emotion classification. The plot reveals that lower frequency bands (0-1Hz, 1-2Hz) contribute most significantly to the classification, aligning with the expected effective frequency range of pulse signals. This result suggests that the model's classification is grounded in relevant physiological signals, rather than biased by noise or unrelated features.

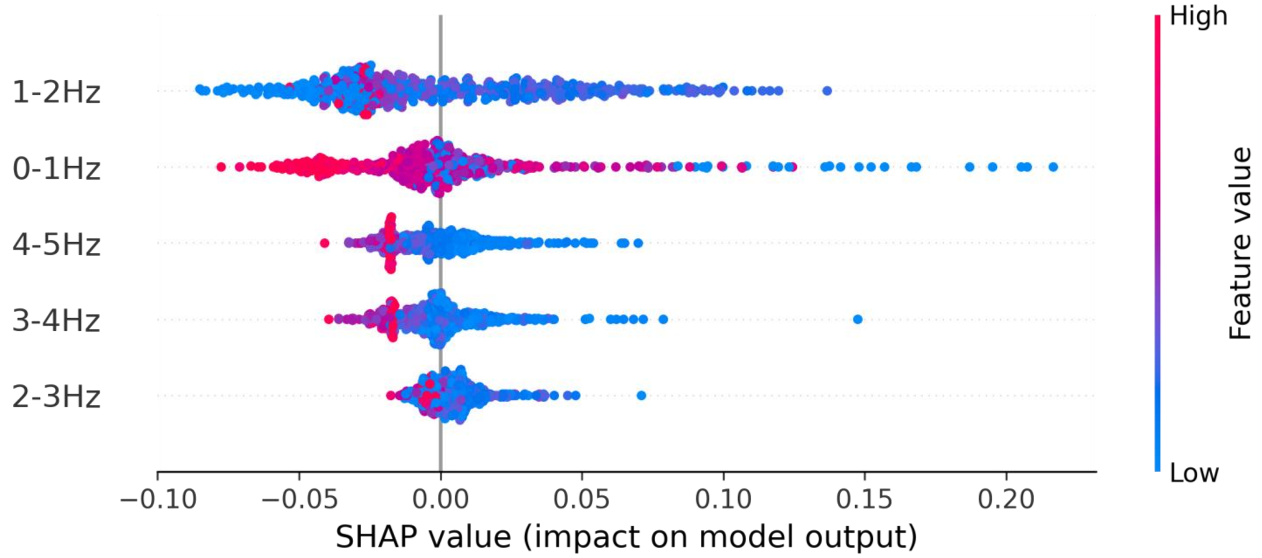

Supplementary Figure 12: Demonstration of some SEA expansion results, comparing basic expansion, CoT prompt expansion, and the combined result of CoT prompt and few-shot demo prompt. The original results were in Chinese and have been translated into English. It can be observed that the CoT prompt effectively enhances the sentence's coherence, and when combined with the few-shot demo, the sentences better align with the patients' usual language habits (such as being gentle, straightforward, polite, etc.).

Example 1: Expanding the Sentence "Let's go to eat"

Emotion Label: Neutral

Objective Information: 22 ℃, 12:30 PM, Sunny

Basic Prompt

Prompt: Given a [neutral] tone and the objective information [22 ℃, 12:30 PM, sunny], expand [Let's go to eat] to a complete sentence.

Result: "Let's go have something to eat in this good day."

CoT Prompt

Prompt: Using the [neutral] tone, with objective information that it's [22 ℃, 12:30 PM, and sunny], expand [Let's go to eat] to a friendly, inviting sentence that reflects the pleasant weather and midday timing. Make the suggestion sound relaxed and casual, as one would say when stepping out for lunch on a nice day.

Result: "It's a beautiful day out, and just about lunchtime. How about we go grab something to eat?"

CoT + Few-shot Learning Prompt

Prompt: ******[CoT prompt]. Please also consider the user's preference of conversation demonstrated in the following examples ****** [personalized examples]

Result: "It's such a sunny day, and it's around lunchtime. Shall we head out for a bite to eat?"

Example 2: Expanding the Sentence "I want to drink water"

Emotion Label: Relieved

Objective Information: 20 ℃, 3:00 PM, Partly cloudy

Basic Prompt

Prompt: Given a [relieved] tone and objective information [20 ℃, 3:00 PM, partly cloudy], expand [I want to drink water] to a complete sentence.

Result: "I'd like to have some water."

CoT Prompt

Prompt: Using a [relieved] tone, with objective information that it's [20 ℃, 3:00 PM,] and partly cloudy, expand [I want to drink water] into a sentence that reflects a sense of relaxation. Make the request sound casual, as though it's part of a restful afternoon.

Result: "It's been a long day, and I finally feel like I can relax. A glass of water would be perfect right now."

CoT + Few-shot Learning Prompt

Prompt: ******[CoT prompt]. Please also consider the user's preference of conversation demonstrated in the following examples ****** [personalized examples]

Result: "It's a bit of a warm afternoon, and I'm feeling better now. Could I have a glass of water to refresh?"

Example 3: Expanding the Sentence "I am tired"

Emotion Label: Neutral

Objective Information: 16 ℃, 7:00 PM, Raining

Basic Prompt

Prompt: Given a [neutral] tone and objective information [16 ℃, 7:00 PM, raining], expand [I am tired] to a complete sentence.

Result: "I feel tired in this rainy day."

CoT Prompt

Prompt: Using a [neutral] tone, with objective information that it's [16 ℃, 7:00 PM, and raining], expand [I am tired] into a sentence that conveys a sense of winding down for the day. Make it sound natural, as one might say after a long day when it's rainy and peaceful outside.

Result: "It's been a long day, and with the rain outside, I feel like winding down soon."

CoT + Few-shot Learning Prompt

Prompt: ******[CoT prompt]. Please also consider the user's preference of conversation demonstrated in the following examples ****** [personalized examples]

Result: "It's raining and getting late. I think I'll rest soon if that's okay."

Supplementary Figure 13: Scores for SEA-generated sentences by different patient subjects.

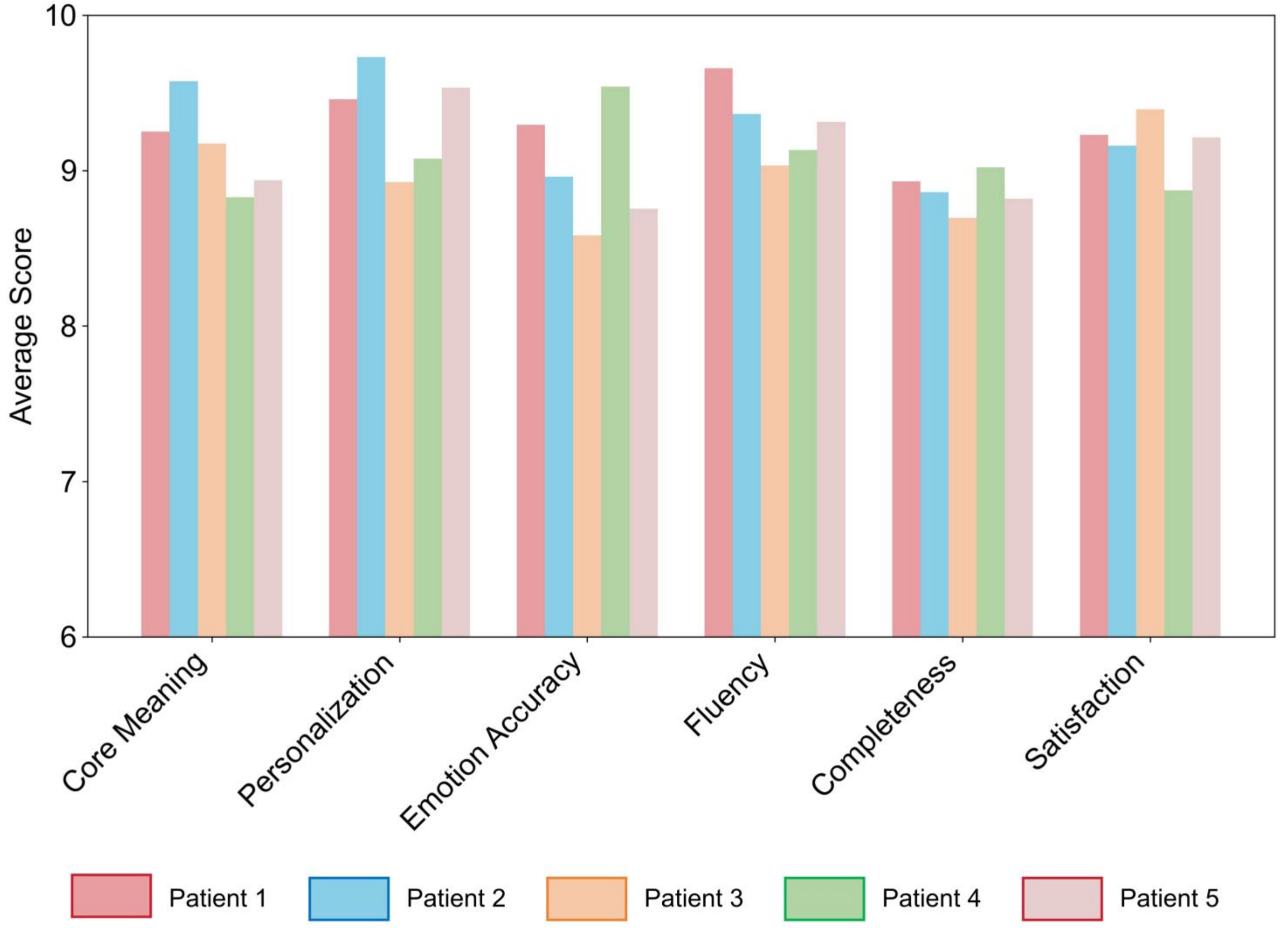

Supplementary Figure 14: Real-time system latency distribution (silent speech to playback). The box represents the interquartile range (25th–75th percentiles), the center line indicates the median, whiskers extend to 1.5× the interquartile range, and points denote outliers.

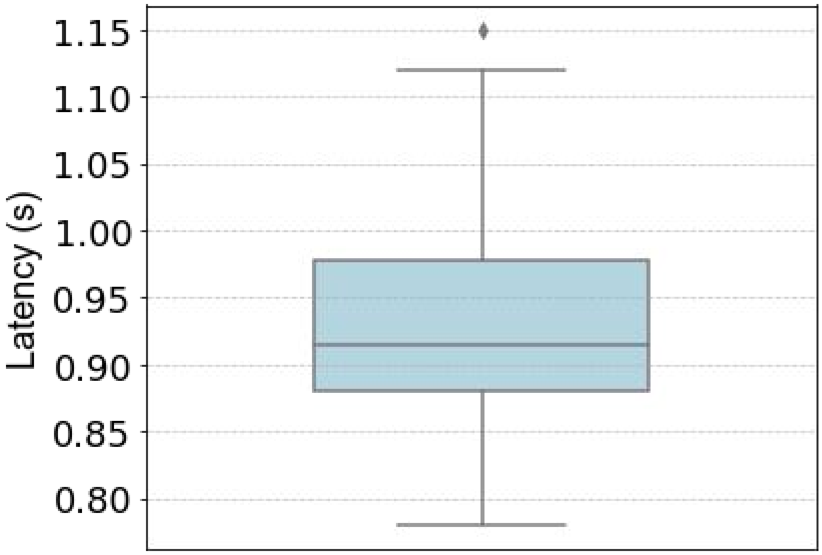

Supplementary Figure 15: Assembly and wearing configuration of the signal acquisition system. **a**, Signal acquisition device. **b**, Installation of the device's bottom enclosure. **c**, Connection of the signal acquisition contacts to the external circuit using copper foil tape. **d**, Copper foil tape connects the contacts to the bottom side, facilitating subsequent integration with the sensing elements. **e**, Front view of the fully assembled device. **f**, Side view of the fully assembled device. **g**, Installation of the sensing elements. **h**, Sensing elements connected laterally via conductive yarns, with reserved length for interfacing with the signal acquisition device. **i**, Connection of the conductive yarns to the copper foil tape at the device bottom and fixation of the device onto the textile band. **j**, Side view of the device worn on the neck. **k**, Frontal view of the device worn on the neck.

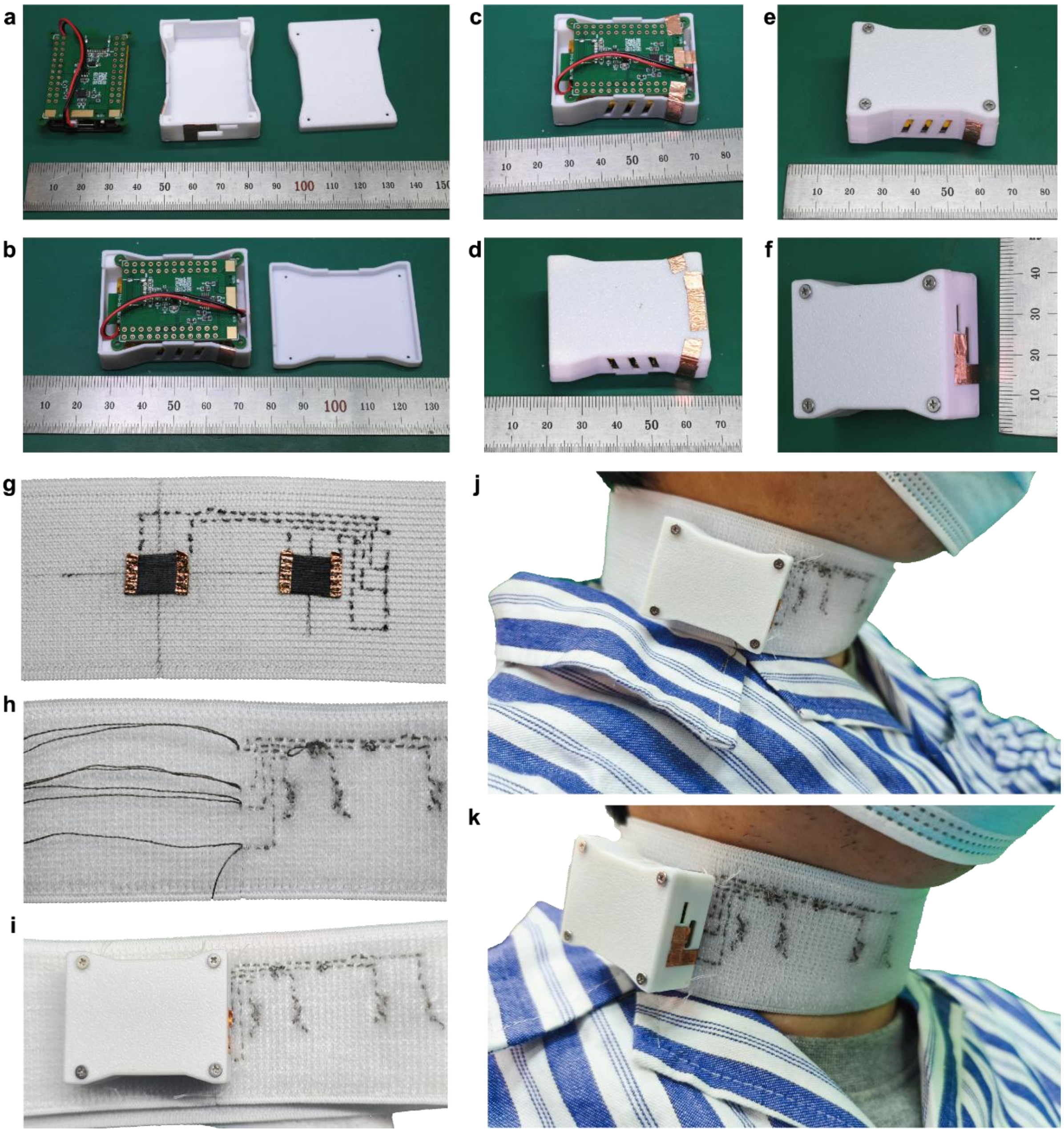

Supplementary Figure 16: Pairwise confusion analysis for viseme-similar word pairs.

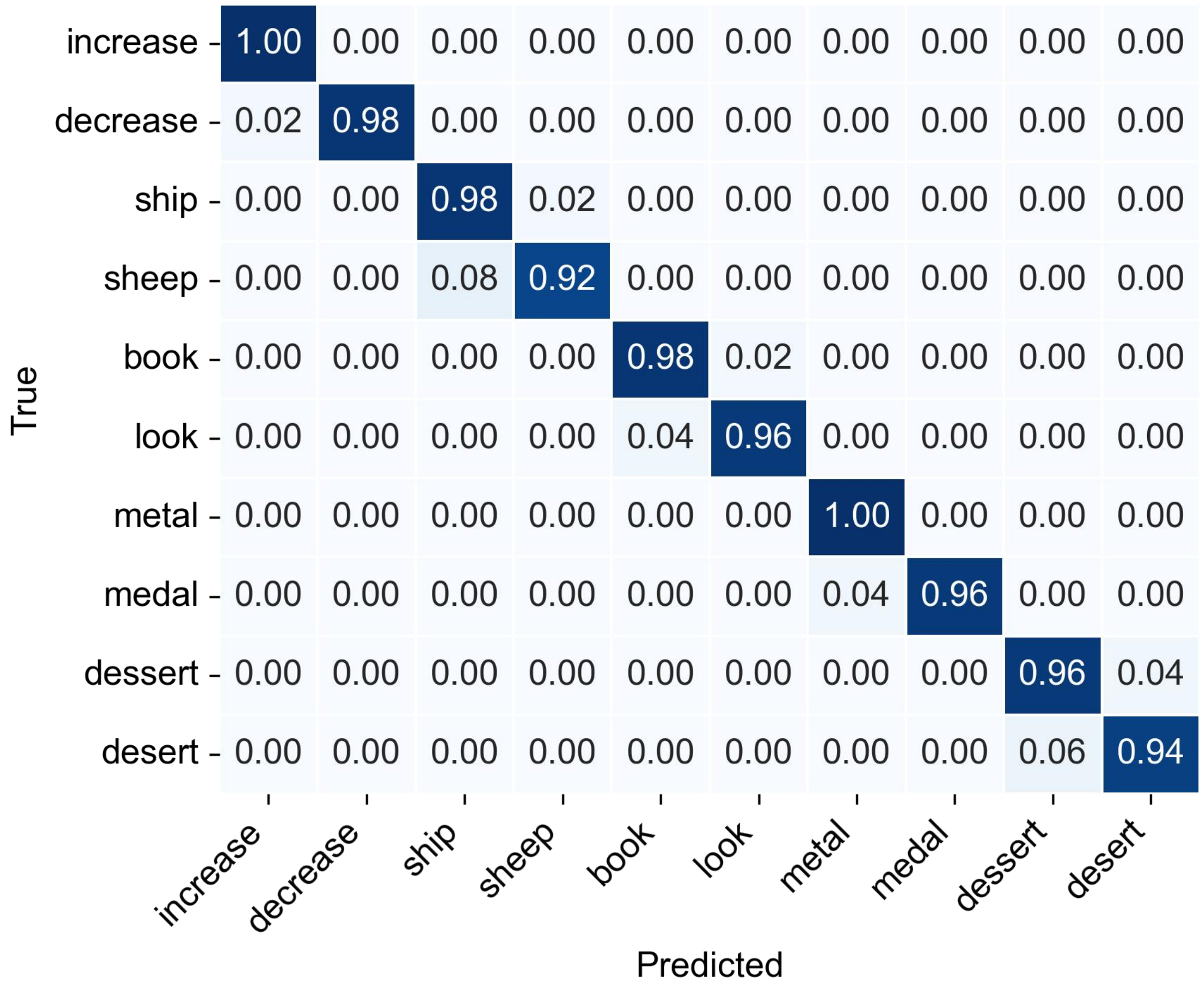

Fig. S17: Raw waveform patterns and relevance-weighted activation maps (Grad-CAM) for four viseme-similar word pairs. [20]

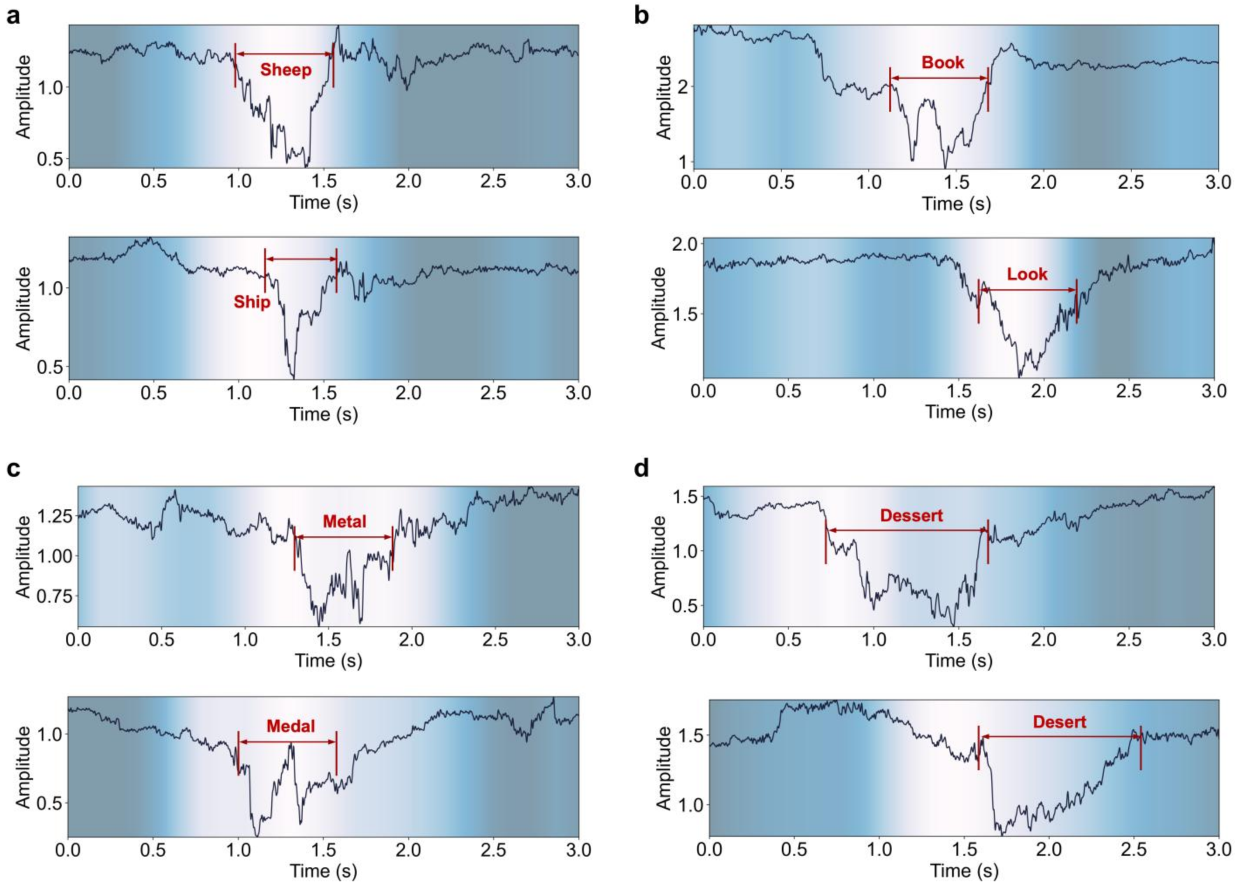

Supplementary Figure 18: Cyclic durability of the printed textile strain sensor. a, Relative resistance variation of a 9 mm × 3 mm printed strip under 0.1–1% uniaxial tensile cycling at 1 Hz for 5000 cycles. **b**, Relative resistance variation of an 18 mm × 6 mm printed strip under cyclic bending at 1 Hz for 5000 cycles with a minimum bending radius of ~5 mm.

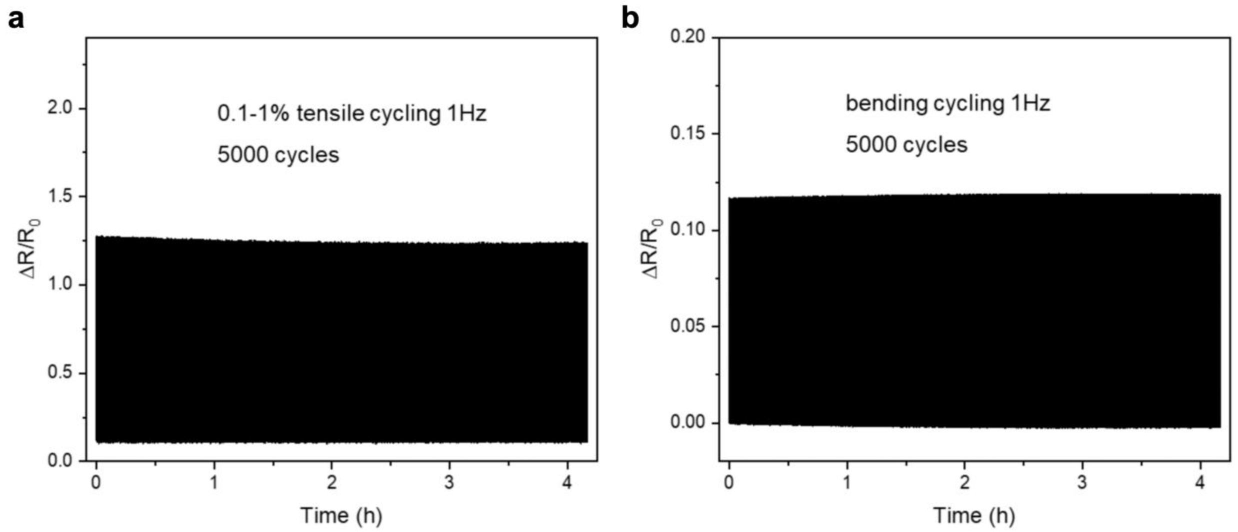

Supplementary Figure 19: Frequency-dependent strain response behavior of the printed textile strain sensor.

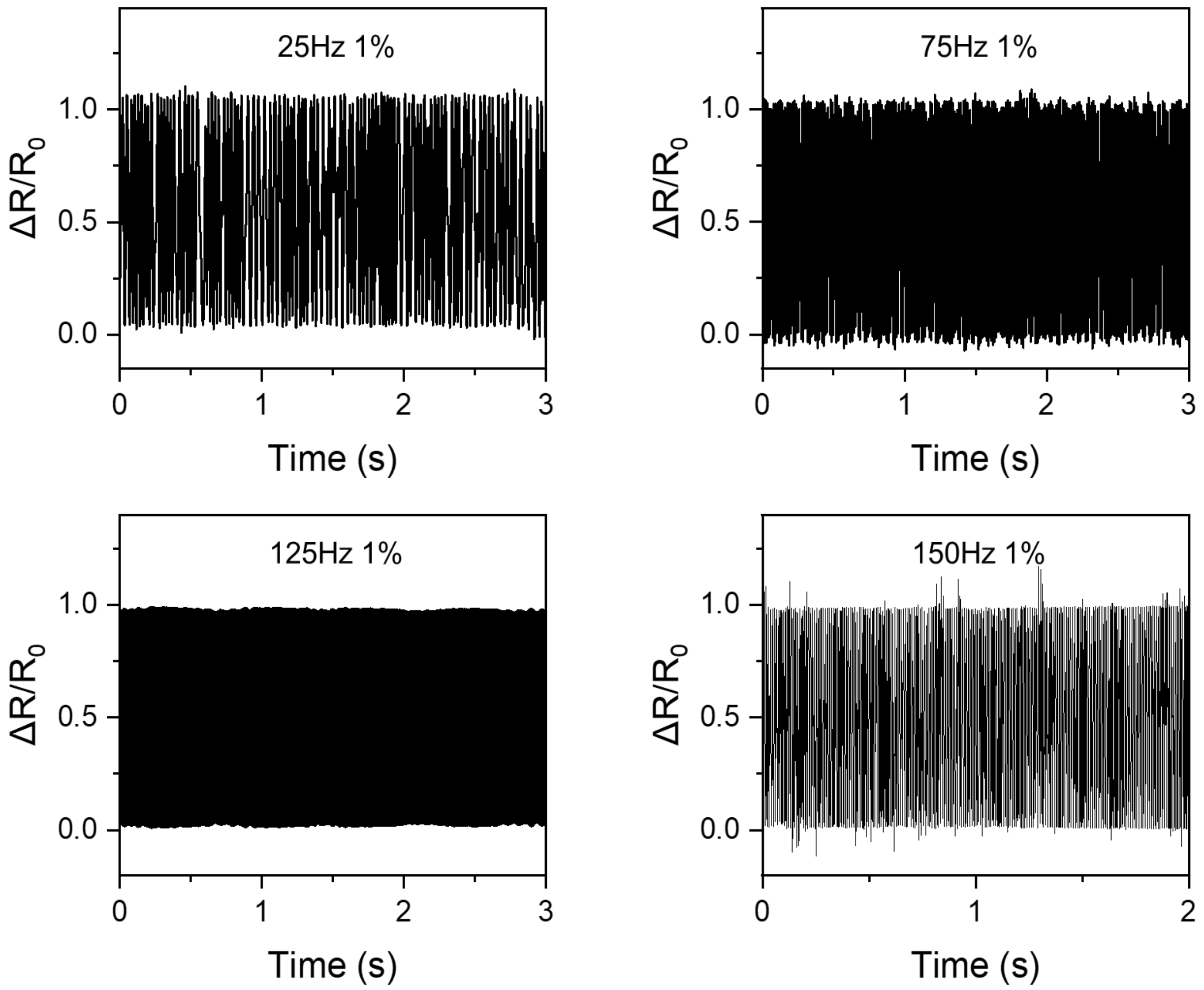

Supplementary Figure 20: Anisotropic strain-response behavior of IT. The x-axis is defined as the longitudinal direction of the strip sample. **a,** Strain response to x-axis stretching. **b,** Strain response along the x-axis during y-axis stretching. **c,** Compressive response to z-axis pressure. The blue shaded area indicates the tension range of normal wear.

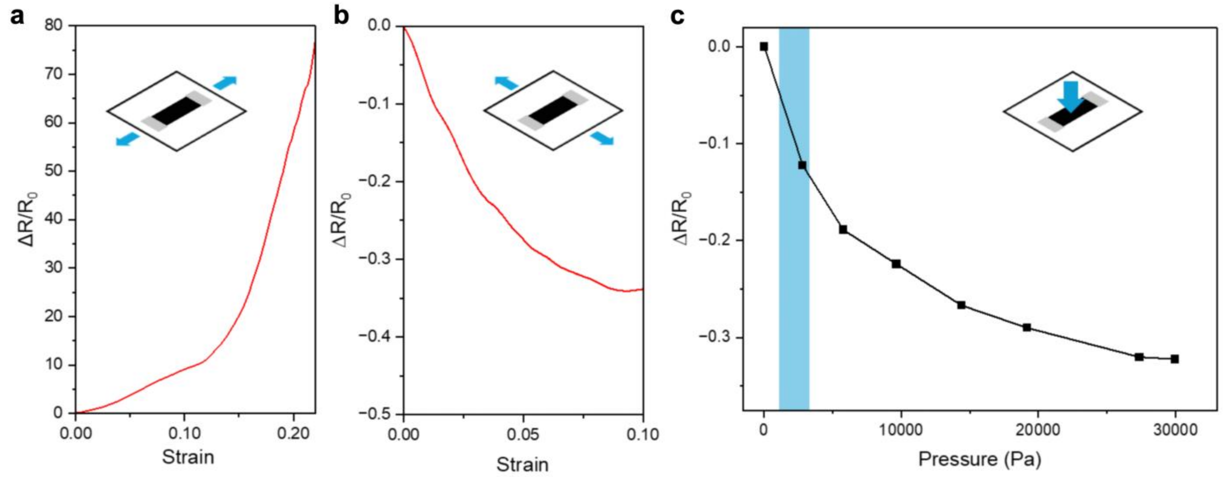

Supplementary Figure 21: Comparison of the strain response of IT with/without strain isolation layer (SIL).

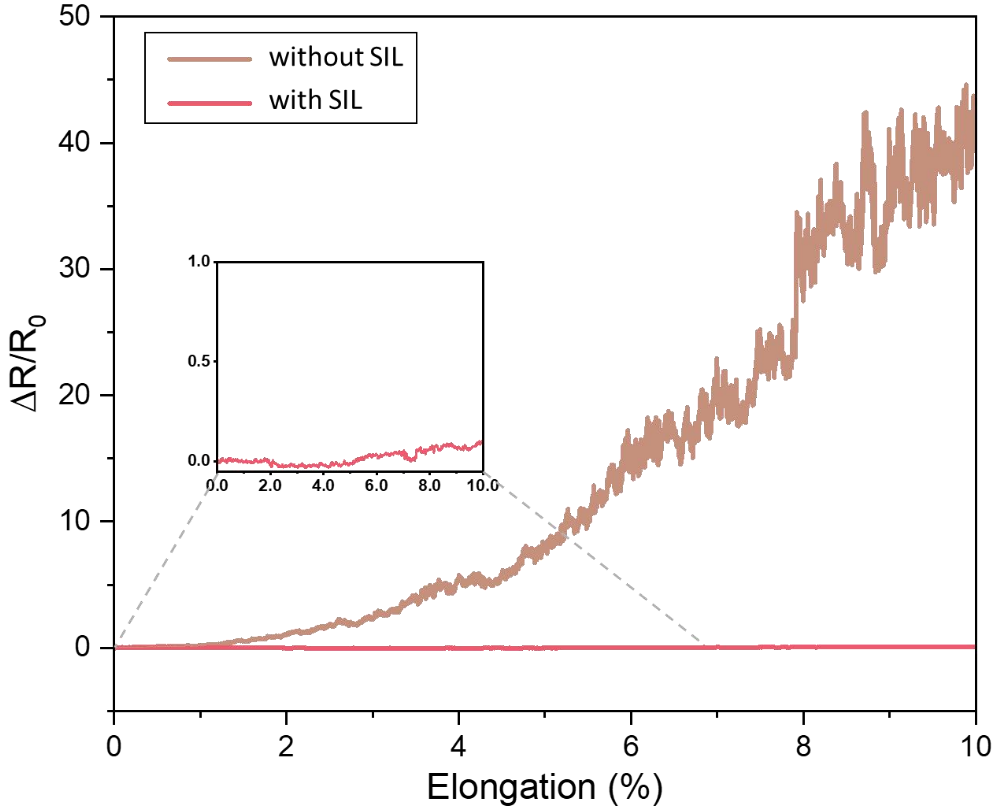

Supplementary Table 1: Comparison of the proposed intelligent throat's features with state-of-the-art wearable silent speech systems.

	**This Work**	Yang *et al.* [11]	Liu *et al.* [12]	Kim *et al.* [13]	Tang *et al.* [14]	Lu *et al.* [15]
Form factor	Textile choker	Throat patch	Headband+ facial unit	Facial patches	Textile choker	Mask
Sensors	Two strain sensors	One strain sensor	Three IMUs	Eight strain sensors	One strain sensor	One TENG sensor
Sensing materials	Graphene	Laser-scribed graphene	Platinum	Crystalline-silicon	Graphene	PVC and nylon
Corpus	47 words, 20 sentences	Four tones, five vowels, and six words	93 words, 92 sentences	100 words	30 words	20 words
Subjects	10 healthy individuals, five stroke patients	Five healthy individuals, one post-laryngectomy patient	Eight healthy individuals	Two healthy individuals	Six healthy individuals	Four healthy individuals
Performance summary	Word error rate of 4.2%, sentence error rate of 2.9%, and overall satisfaction rated as "fully satisfied" on five stroke patients	Average accuracy of 99.05% on healthy individuals, and 91% accuracy on one post-laryngectomy patient	Word accuracy of 97.4%, sentence accuracy of 92.0%	Word accuracy of 87.53%	Word accuracy of 95.25%	Word accuracy of 94.5%
Zero-delay expression	√	×	×	×	×	×
Emotive-logical capacity	√	×	×	×	×	×
Wireless	√	×	√	×	×	√
Robustness[1]	Good	Medium	Good	Medium	Medium	Medium
Comfortability[2]	Good	Medium	Medium	Bad	Medium	Medium
Patients accessibility	Good	Medium	Bad	Bad	Bad	Bad

[1]Robustness is a comprehensive measure, including durability, resistance to artefacts, and environmental stability.
[2]Comfortability is a comprehensive measure, including stretchability, biocompatibility, and breathability.

Supplementary Table 2: Corpus words list.

ID	Chinese Vocabulary	English Translation	ID	Chinese Vocabulary	English Translation
1	我	I	25	洗澡	Take a shower
2	我的	My	26	厕所	Toilet
3	我们	We	27	一个	One
4	你	You	28	这些	These
5	想要	Want	29	多少	How much
6	需要	Need	30	钱	Money
7	吃饭	Eat	31	你好	Hello
8	睡觉	Sleep	32	谢谢	Thank you
9	医院	Hospital	33	请	Please
10	去	Go	34	麻烦	Excuse me
11	哪里	Where	35	今天	Today
12	回来	Come back	36	明天	Tomorrow
13	什么	What	37	胳膊	Arm
14	时候	When	38	很好	Very good
15	眼睛	Eyes	39	不用	Don’t need
16	舒服	Comfortable	40	担心	worry
17	不	Not	41	杯子	Cup
18	累了	Tired	42	勺子	Spoon
19	好的	Okay	43	筷子	Chopsticks
20	知道	Know	44	水果	Fruit
21	你们	You (plural)	45	电话	Phone
22	我想	I want	46	微信	WeChat (messaging app)
23	给我	Give me	47	联系	Contact
24	喝水	Drink Water			

Supplementary Table 3: Corpus sentences list.

ID	Chinese Sentence	English Translation
1	我们去吃饭	Let’s go to eat
2	我想要喝水	I want to drink water
3	我需要洗澡	I need to take a shower
4	我想去厕所	I want to go to the toilet
5	我想睡觉	I want to sleep
6	我需要去医院	I need to go to the hospital
7	你们去哪里	Where are you (plural) going?
8	你今天什么时候回来	When are you coming back today?
9	我们明天去医院	Let’s go to the hospital tomorrow
10	我的眼睛不舒服	My eyes are uncomfortable
11	我胳膊不舒服	My arm is uncomfortable
12	我很好，不用担心	I am fine, no need to worry
13	我累了	I am tired
14	好的，我知道	Okay, I know
15	你好，请给我一个杯子	Hello, please give me a cup
16	麻烦给我一个勺子	Please give me a spoon
17	谢谢你	Thank you
18	这些水果多少钱	How much are these fruits?
19	我们微信联系	Let’s contact via WeChat
20	你电话多少	What is your phone number?

Supplementary Table 4: Scoring criteria used for evaluating the generated sentences.

Criterion	Description	Scoring Guidelines
**Core Meaning Expression**	Does the sentence accurately express the intended core meaning?	1-3: Sentence fails to convey the core meaning.<br>4-6: Partially conveys the meaning.<br>7-8: Mostly accurate.<br>9-10: Fully accurate.
**Personalization & Habit Matching**	Does the sentence align with the patient's usual speaking habits and style?	1-3: Does not match at all.<br>4-6: Partially matches the patient's style.<br>7-8: Mostly matches.<br>9-10: Fully aligned with patient's usual speech.
**Emotion Expression Accuracy**	Is the generated sentence reflecting the correct emotional tone as intended?	1-3: Incorrect emotion.<br>4-6: Partially correct emotion.<br>7-8: Mostly correct emotion.<br>9-10: Emotion fully accurate.
**Sentence Naturalness & Fluency**	Is the sentence natural and fluent, similar to everyday conversation?	1-3: Very unnatural and stiff.<br>4-6: Somewhat natural but still mechanical.<br>7-8: Mostly natural.<br>9-10: Completely fluent and natural.
**Completeness of Generated Sentence**	Is the sentence complete, with no important missing information?	1-3: Major information missing.<br>4-6: Some details missing.<br>7-8: Mostly complete.<br>9-10: Fully complete, no missing details.
**Overall User Satisfaction**	How satisfied is the user with the generated sentence?	1-3: Very dissatisfied.<br>4-6: Somewhat satisfied.<br>7-8: Mostly satisfied.<br>9-10: Fully satisfied.

Supplementary Table 5: Long-term performance test results.

Patient	WER (Initial, %)	WER (After 6 Months, %)	WER (After Fine-tuning, %)
**1**	3.8	9.5	3.9
**2**	4.5	10.8	4.4
**3**	4.1	10.3	4.2
**4**	4.3	9.9	4.1
**5**	4.2	10.2	4.5

Supplementary Table 6: Patient comfortability rating. Each patient rated the system on four key aspects—weight, fit, material flexibility, and long-term wearability—on a 1 to 5 scale (1 = very uncomfortable, 5 = very comfortable).

**Patient**	**Weight Comfort**	**Fit Comfort**	**Long-Term Wearability**	**Flexibility**
**1**	4	5	3	4
**2**	3	4	4	4
**3**	4	4	4	4
**4**	5	5	4	5
**5**	4	4	4	4

Supplementary Table 7: Patient communication evaluation on a 1 to 5 scale (1 = very uncomfortable, 5 = very comfortable).

Patient	Conversational Efficiency	Listener Comprehensiveness	Communication Satisfaction
**1**	4	5	4
**2**	4	5	5
**3**	4	5	4
**4**	5	5	4
**5**	4	5	5

Supplementary Table 8: Overview of dysarthria severity levels in enrolled patients.

Patient	Age	Gender	Severity Level	Speech Characteristics
**1**	34	Male	Mild	Imprecise articulation, reduced speaking rate
**2**	47	Male	Moderate	Inconsistent phonation, mild hypernasality
**3**	56	Male	Mild	Mild articulation difficulty, slow speech
**4**	41	Male	Moderate	Moderate slurred speech, irregular rhythm
**5**	37	Female	Mild	Mild prosodic abnormalities, slightly strained voice

[1]Severe dysarthria patients who have lost neuromuscular ability are not accessible to this technology.

Supplementary Table 9: Quantitative comparison of the proposed IT system with alternative sensing modalities under identical placement and silent speech protocols (direct synthesis mode).

Sensor type	Word error rate (%)	Comfort rating	Remarks
Textile strain sensor (this work)	4.2	High	Flexible, skin-conformal
MEMS accelerometer	27.6	Low	Rigid, motion-sensitive
PVDF film	10.3	Medium	Fragile under strain

Supplementary References

[1] Pinto, S., *et al.* Treatments for dysarthria in Parkinson's disease. *The Lancet Neurology* **3**, 547-556 (2004).

[2] Noffs, G., *et al.* What speech can tell us: A systematic review of dysarthria characteristics in Multiple Sclerosis. *Autoimmunity reviews* **17**, 1202-1209 (2018).

[3] GBD 2019 Stroke Collaborators. Global, regional, and national burden of stroke and its risk factors, 1990–2019: a systematic analysis for the Global Burden of Disease Study 2019. *The Lancet. Neurology* **20**, 795-820 (2021).

[4] Enderby, P. Disorders of communication: dysarthria. *Handbook of clinical neurology* **110**, 273-281 (2013).

[5] Zinn, S., *et al.* The effect of poststroke cognitive impairment on rehabilitation process and functional outcome. *Archives of physical medicine and rehabilitation* **85**, 1084-1090 (2004).

[6] Teshaboeva, F. Literacy education of speech impaired children as a pedagogical psychological problem." *Confrencea* **5**, 299-302 (2023).

[7] Beaulieu, C. L., *et al.* Occupational, physical, and speech therapy treatment activities during inpatient rehabilitation for traumatic brain injury. *Archives of physical medicine and rehabilitation* **96**, 222-234 (2015).

[8] Karges, J., and Smallfield, S. A description of the outcomes, frequency, duration, and intensity of occupational, physical, and speech therapy in inpatient stroke rehabilitation. *Journal of allied health* **38**, 1-10 (2009).

[9] Patrick-Krueger, K.M., Burkhart, I. & Contreras-Vidal, J.L. The state of clinical trials of implantable brain–computer interfaces. *Nature Reviews Bioengineering* (2024).

[10] Silva, A. B., Littlejohn, K. T., Liu, J. R., Moses, D. A. & Chang, E. F. The speech neuroprosthesis. *Nature Reviews Neuroscience* **25**, 473–492 (2024).

[11] Yang, Q. *et al.* Mixed-modality speech recognition and interaction using a wearable artificial throat. *Nature Machine Intelligence* **5**, 169–180 (2023).

[12] Liu, S., *et al.* A data-efficient and easy-to-use lip language interface based on wearable motion capture and speech movement reconstruction. *Science Advances* **10**, eado9576 (2024).

[13] Kim, T., *et al*. Ultrathin crystalline-silicon-based strain gauges with deep learning algorithms for silent speech interfaces. *Nature Communications* **13**, 5815 (2022).

[14] Tang, C. *et al.* Ultrasensitive textile strain sensors redefine wearable silent speech interfaces with high machine learning efficiency. *npj Flexible Electronics* **8**, 27 (2024).

[15] Lu, Y., *et al.* Decoding lip language using triboelectric sensors with deep learning. *Nature Communications* **13**, 1401 (2022).

[16] Ma, C. *et al.* High sensitivity, broad working range, comfortable, and biofriendly wearable strain sensor for electronic skin. *Advanced Materials Technologie*s **7**, 2200106 (2022).

[17] Smilkov, D., *et al.* SmoothGrad: removing noise by adding noise. *arXiv:1706.03825.* (2017).

[18] Tang, C. *et al.* A deep learning-enabled smart garment for accurate and versatile sleep conditions monitoring in daily life. *The Proceedings of the National Academy of Sciences (PNAS)* **122**, e2420498122 (2025).

[19] Ahmed, T. and Devanbu, P. Better patching using llm prompting, via self-consistency. In *2023 38th IEEE/ACM International Conference on Automated Software Engineering (ASE)*, 1742-1746 (2023)

[20] Selvaraju, R. R. *et al.* Grad-cam: Visual explanations from deep networks via gradient-based localization. In *Proceedings of the IEEE International Conference on Computer Vision*, 618-626 (2017).
