# Supplementary material for: Wearable intelligent throat enables natural speech in stroke patients with dysarthria
Source: Nat Commun. 2026 Jan 19;17:293. doi: 10.1038/s41467-025-68228-9 (PMC12816716; doi:10.1038/s41467-025-68228-9)
Supplement: Supplementary file 2 — Description of Additional Supplementary Files [file 41467_2025_68228_MOESM2_ESM.pdf]

Description of Additional Supplementary Files

File Name: Supplementary Video 1

Description: Stroke patient with dysarthria attempts to speak.

This video shows the scenes of a stroke patient with dysarthria engaging in word/sentence echoic training and simple daily conversation exercises with a rehabilitation therapist, without IT assistance.

File Name: Supplementary Video 2

Description: Intelligent Throat: system overview.

This video illustrates the functional workflow of each component within the IT system during operation.

File Name: Supplementary Video 3

Description: Intelligent Throat: live demonstration.

This video showcases the real-world use case of the wearable IT system in practical scenarios.
