# Supplementary material for: Wearable intelligent throat enables natural speech in stroke patients with dysarthria
Source: Nat Commun. 2026 Jan 19;17:293. doi: 10.1038/s41467-025-68228-9 (PMC12816716; doi:10.1038/s41467-025-68228-9)
Supplement: Supplementary file 6 — Reporting Summary [file 41467_2025_68228_MOESM6_ESM.pdf]

nature portfolio

Corresponding author(s): Shuo Gao, Luigi G. Occhipinti

Last updated by author(s): Dec 16, 2025

Reporting Summary

Nature Portfolio wishes to improve the reproducibility of the work that we publish. This form provides structure for consistency and transparency in reporting. For further information on Nature Portfolio policies, see our Editorial Policies and the Editorial Policy Checklist.

Statistics

For all statistical analyses, confirm that the following items are present in the figure legend, table legend, main text, or Methods section.

n/a	Confirmed	
☐	☒	The exact sample size (*n*) for each experimental group/condition, given as a discrete number and unit of measurement
☐	☒	A statement on whether measurements were taken from distinct samples or whether the same sample was measured repeatedly
☐	☒	The statistical test(s) used AND whether they are one- or two-sided<br>*Only common tests should be described solely by name; describe more complex techniques in the Methods section.*
☐	☒	A description of all covariates tested
☒	☐	A description of any assumptions or corrections, such as tests of normality and adjustment for multiple comparisons
☒	☐	A full description of the statistical parameters including central tendency (e.g. means) or other basic estimates (e.g. regression coefficient) AND variation (e.g. standard deviation) or associated estimates of uncertainty (e.g. confidence intervals)
☒	☐	For null hypothesis testing, the test statistic (e.g. *F*, *t*, *r*) with confidence intervals, effect sizes, degrees of freedom and *P* value noted<br>*Give P values as exact values whenever suitable.*
☒	☐	For Bayesian analysis, information on the choice of priors and Markov chain Monte Carlo settings
☒	☐	For hierarchical and complex designs, identification of the appropriate level for tests and full reporting of outcomes
☒	☐	Estimates of effect sizes (e.g. Cohen's *d*, Pearson's *r*), indicating how they were calculated

Our web collection on statistics for biologists contains articles on many of the points above.

Software and code

Policy information about availability of computer code

Data collection	Data were collected using a custom-built wearable system consisting of textile strain sensors and a custom-designed PCB for silent speech and carotid pulse signal acquisition. Signal acquisition was performed using in-house firmware developed for the STM32G431 microcontroller (STM32CubeIDE v1.12.0), and data were transmitted via a BLE module (BLE-SER-A-ANT) using UART protocol. Data recording and synchronization were managed via a custom Python 3.8.13 script running on a local server. No commercial or open-source GUI-based acquisition platforms were used.
Data analysis	Data analysis was performed using custom Python scripts (Python 3.8.13) with the following open-source packages: PyTorch 2.0.1 for model training and inference; NumPy 1.23.4 and SciPy 1.9.3 for signal processing and statistical analysis; scikit-learn 1.1.3 for classifier benchmarking and SHAP value analysis; matplotlib 3.6.2 and seaborn 0.12.1 for data visualization; UMAP 0.5.3 for dimensionality reduction. All analysis was run on a Linux-based system with CUDA 11.7 and NVIDIA A100 GPU acceleration. No commercial software was used.

For manuscripts utilizing custom algorithms or software that are central to the research but not yet described in published literature, software must be made available to editors and reviewers. We strongly encourage code deposition in a community repository (e.g. GitHub). See the Nature Portfolio guidelines for submitting code & software for further information.

Data

Policy information about availability of data

All manuscripts must include a data availability statement. This statement should provide the following information, where applicable:

- Accession codes, unique identifiers, or web links for publicly available datasets
- A description of any restrictions on data availability
- For clinical datasets or third party data, please ensure that the statement adheres to our policy

The datasets generated and analyzed during the current study, including silent speech signals, carotid pulse recordings, and token-level labels from both healthy participants and stroke patients with dysarthria, will be made available via a public GitHub repository upon publication. During peer review, the full dataset will be made available to editors and reviewers via a private GitHub repository link. All data are de-identified and comply with institutional ethics requirements (IRB approval number: 2023HL-142-01).

Research involving human participants, their data, or biological material

Policy information about studies with human participants or human data. See also policy information about sex, gender (identity/presentation), and sexual orientation and race, ethnicity and racism.

Reporting on sex and gender	Sex (biological attribute) of participants was collected and reported. The healthy subject group (n = 10) included 6 males and 4 females. The patient group (n = 5, stroke patients with dysarthria) included 4 males and 1 female. Sex was self-reported by participants during enrollment. Gender identity was not collected, and no gender-based analysis was performed.<br><br>Sex was not considered as a variable during model training or evaluation due to the limited sample size, and because the study's primary focus was on signal decoding performance across individuals with dysarthria, regardless of sex. Future studies with larger, more balanced cohorts may explore potential sex-based physiological signal differences.
Reporting on race, ethnicity, or other socially relevant groupings	All participants (healthy subjects and stroke patients) self-identified as ethnically Chinese. Race or ethnicity was not used as a variable in study design, analysis, or interpretation. Healthy subjects were recruited from Beihang University (Beijing, China), and stroke patients were recruited from hospitals in Henan province. No socially constructed groupings were used to categorize or stratify data. Given the limited demographic diversity and the study's focus on neuromuscular signal decoding, race and ethnicity were not analyzed as confounding variables.
Population characteristics	The study included 10 healthy adult participants (6 males, 4 females; mean age 25.3 ± 4.1 years) recruited from Beihang University, and 5 stroke patients with dysarthria (4 males, 1 female; mean age 43.0 ± 7.8 years) recruited from rehabilitation hospitals in Henan, China. All participants self-identified as Chinese. Stroke patients were diagnosed with dysarthria following ischemic stroke and retained partial laryngeal motor control, confirmed by clinical assessment. No participants had concurrent neurological or psychiatric disorders. All participants provided informed consent prior to participation.
Recruitment	Healthy participants were recruited via internal advertisements at Beihang University. Stroke patients with dysarthria were recruited from rehabilitation centers in Henan based on physician referrals and voluntary participation. All participants provided written informed consent. Potential self-selection bias may exist due to voluntary enrollment, especially among the healthy group who were university students. However, the primary aim was to evaluate signal decoding in stroke patients, and data from healthy subjects were used for pretraining only.
Ethics oversight	The study protocol was reviewed and approved by the Ethics Committee of the First Affiliated Hospital of Henan University of Chinese Medicine (approval number: 2023HL-142-01). All methods were carried out in accordance with relevant guidelines and regulations.

Note that full information on the approval of the study protocol must also be provided in the manuscript.

Field-specific reporting

Please select the one below that is the best fit for your research. If you are not sure, read the appropriate sections before making your selection.

☒ Life sciences ☐ Behavioural & social sciences ☐ Ecological, evolutionary & environmental sciences

For a reference copy of the document with all sections, see nature.com/documents/nr-reporting-summary-flat.pdf

Life sciences study design

All studies must disclose on these points even when the disclosure is negative.

Sample size	The sample size was not statistically predetermined. Instead, it was based on prior studies of silent speech decoding in clinical populations and feasibility constraints associated with recruiting stroke patients with dysarthria. Ten healthy participants were used to pretrain the model, and five stroke patients were recruited for model fine-tuning and evaluation. Each participant contributed multiple repetitions per word and sentence (up to 100 for healthy subjects and 50 for patients), resulting in a sufficiently large number of token-level samples (>10,000 per group) for machine learning purposes. This sample size was deemed adequate to demonstrate proof-of-concept performance in a real-world assistive setting.
Data exclusions	No participants were excluded from the study. Data exclusion was limited to rare instances of sensor disconnection or signal acquisition

	failure, where no measurable signal was recorded. These exclusion criteria were pre-established and applied only when signals exhibited complete dropout (i.e., flatline signal with zero variance). All other data, including those with noise or minor motion artifacts, were retained to support real-world generalization.
Replication	All experiments were performed with multiple repetitions per participant (up to 100 trials per word for healthy subjects and 50 for patients). Each model was trained and evaluated using independently collected data samples. In the follow-up test conducted six months later, a subset of patients repeated the experiment, and the model retained performance after few-shot fine-tuning, confirming temporal reproducibility. No replication failures were observed.
Randomization	Participants were not randomized into groups as the study employed a within-group design. Healthy subjects were used for model pretraining, and stroke patients were used for fine-tuning and evaluation. Group allocation was based on clinical status (healthy vs. dysarthria) and not influenced by experimental variables. Randomization was not relevant to this machine-learning-focused study.
Blinding	Blinding was not applicable. All data were collected and labeled based on participants' self-expressed silent speech and known word prompts during supervised sessions. Since the goal was to decode known speech tokens and emotions using sensor data, investigators were aware of ground truth during training and evaluation. The nature of the study did not permit or require blinding.

Reporting for specific materials, systems and methods

We require information from authors about some types of materials, experimental systems and methods used in many studies. Here, indicate whether each material, system or method listed is relevant to your study. If you are not sure if a list item applies to your research, read the appropriate section before selecting a response.

Materials & experimental systems

n/a	Involved in the study
☒	☐ Antibodies
☒	☐ Eukaryotic cell lines
☒	☐ Palaeontology and archaeology
☒	☐ Animals and other organisms
☐	☒ Clinical data
☒	☐ Dual use research of concern
☒	☐ Plants

Methods

n/a	Involved in the study
☒	☐ ChIP-seq
☒	☐ Flow cytometry
☒	☐ MRI-based neuroimaging

Clinical data

Policy information about clinical studies

All manuscripts should comply with the ICMJE guidelines for publication of clinical research and a completed CONSORT checklist must be included with all submissions.

Clinical trial registration	This study was not a registered clinical trial as defined by ICMJE guidelines. It was a non-interventional observational study focused on evaluating a wearable communication system in stroke patients with dysarthria. The study received institutional ethics approval (see below) and involved no therapeutic interventions or changes to standard of care.
Study protocol	The study protocol was reviewed and approved by the Ethics Committee of the First Affiliated Hospital of Henan University of Chinese Medicine (approval no. 2023HL-142-01). A detailed protocol document can be provided to editors and reviewers upon request.
Data collection	Data collection was conducted between March and September 2024 at the Rehabilitation Center of The First Affiliated Hospital of Henan University of Chinese Medicine. Stroke patients with dysarthria were assessed in a clinical setting under supervision, and silent speech signals and carotid pulse signals were collected using the IT (intelligent throat) wearable device. All data collection adhered to ethical and privacy regulations.
Outcomes	The primary outcome was speech intelligibility and communication fluency using the IT system, assessed by word error rate (WER) and sentence error rate (SER) from LLM-based decoding. The secondary outcome was patient satisfaction and emotional accuracy, assessed through a structured post-session questionnaire with Likert-scale ratings and manual review of emotion-aligned outputs. Performance metrics were compared pre- and post-LLM expansion. No therapeutic outcomes were assessed.

Plants

Seed stocks

Report on the source of all seed stocks or other plant material used. If applicable, state the seed stock centre and catalogue number. If plant specimens were collected from the field, describe the collection location, date and sampling procedures.

Novel plant genotypes

Describe the methods by which all novel plant genotypes were produced. This includes those generated by transgenic approaches, gene editing, chemical/radiation-based mutagenesis and hybridization. For transgenic lines, describe the transformation method, the number of independent lines analyzed and the generation upon which experiments were performed. For gene-edited lines, describe the editor used, the endogenous sequence targeted for editing, the targeting guide RNA sequence (if applicable) and how the editor was applied.

Authentication

Describe any authentication procedures for each seed stock used or novel genotype generated. Describe any experiments used to assess the effect of a mutation and, where applicable, how potential secondary effects (e.g. second site T-DNA insertions, mosiacism, off-target gene editing) were examined.
